# Estimated density of *Borrelia burgdorferi* sensu stricto*-*infected *Ixodes scapularis* nymphs in the eastern United States

**DOI:** 10.1186/s13071-025-06937-2

**Published:** 2025-08-18

**Authors:** Karen M. Holcomb, Erik Foster, Sarah E. Maes, Christina M. Parise, Lynn M. Osikowicz, Andrias Hojgaard, Rebecca J. Eisen

**Affiliations:** Division of Vector Borne Diseases, Centers for Disease Control and Prevention, Fort Collins, CO USA

**Keywords:** Generalized addition model, Acarological risk, Deer ticks, Tick surveillance

## Abstract

**Background:**

Most vector-borne disease cases reported in the United States are caused by pathogens spread by blacklegged ticks, *Ixodes scapularis*. Of these, a majority are Lyme disease cases caused by *Borrelia burgdorferi* sensu stricto (s.s.). Because most human infections are associated with nymphal tick bites, the density of host-seeking *B. burgdorferi s.s.-*infected *I. scapularis* nymphs (DIN) is often used to estimate risk of Lyme disease cases. DIN combines estimates of nymphal infection prevalence with estimates of densities of host-seeking nymphs, making it a costly metric to obtain through tick surveillance. Thus, field-derived estimates of DIN are limited.

**Methods:**

To fill these gaps, we sought to estimate DIN across all counties in the eastern United States. We first estimated *B. burgdorferi* s.s. prevalence in host-seeking *I. scapularis* nymphs using generalized additive models and historical tick surveillance data reported to the Centers for Disease Control and Prevention’s ArboNET database (2004–2023). We then combined prevalence estimates with previously estimated densities of host-seeking nymphs to estimate DIN. We validated these model-based estimates against data reported to ArboNET: field-derived county estimates of prevalence and DIN as well as county records of *B. burgdorferi* s.s. presence and collection of host-seeking nymphs.

**Results:**

We estimated higher average nymphal prevalence (20–30%) in the Upper Midwest and Northeast and lower prevalence (0–5%) throughout the Southeast. Categorizing estimated DIN as minimal or elevated, we identified areas in the Upper Midwest and Northeast as elevated, with the majority of the Southeast and Great Plains as minimal risk.

**Conclusions:**

Our resulting risk map can be used to raise awareness of Lyme disease in communities at elevated risk and aid in the promotion of tick-bite prevention practices.

**Graphical Abstract:**

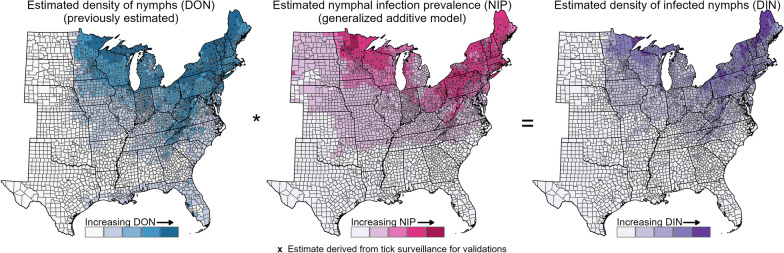

**Supplementary Information:**

The online version contains supplementary material available at 10.1186/s13071-025-06937-2.

## Background

Lyme disease, the most commonly reported vector-borne disease in the United States, is caused primarily by the tick-borne bacterium *Borrelia burgdorferi* sensu stricto (s.s.) [1, 2]. The number and spatial distribution of human Lyme disease cases has continued to increase from historically high incidence foci in the Northeast and Upper Midwest to states in the Ohio Valley and along the Appalachian Mountains in Virginia and North Carolina [2–4]. These increases follow patterns consistent with range expansion of *Ixodes scapularis* and *B. burgdorferi* s.s. [5]. Most cases are associated with bites by *B. burgdorferi* s.s.-infected *I. scapularis* nymphs [6].

In the USA, tick surveillance is used to monitor changes in acarological risk for Lyme and other tick-borne diseases [7]. The US Centers for Disease Control and Prevention’s National Tick Surveillance Program collects data on tick presence and densities as well as pathogen presence and prevalence. A combined metric, the density of *B. burgdorferi* s.s.-infected host-seeking nymphs (DIN), quantifies acarological risk as the product of the density of host-seeking nymphs (DON) and prevalence of *B. burgdorferi* s.s. in host-seeking nymphs (NIP). Previous studies identified significant positive associations between DIN and incidence of Lyme disease at sub-state spatial scales [8, 9]. A previous statistical model based on systematic drag sampling across the eastern USA (2004–2006) showed a significant positive correlation between DIN and Lyme disease incidence across regions in the eastern USA [10, 11]. Geographical changes to the tick’s and pathogen’s reported distributions have been noted during the nearly two decades since these surveillance and modeling efforts occurred [5], highlighting the need for an update. Because DIN is based on extensive field sampling and costly laboratory testing, a limited number of DIN observations are available in the CDC’s tick surveillance database, the ArboNET Tick Module [12].

We sought to estimate DIN in counties across the eastern USA as a means of filling gaps in field-derived observations and estimating acarological risk. Previously, we estimated county-level densities of host-seeking nymphal *I. scapularis* ticks [13]. Here, we developed a generalized additive model (GAM) to generate smoothed county-level estimates of *B. burgdorferi* s.s. prevalence in host-seeking *I. scapularis* nymphs. We then combined these modeled estimates to approximate DIN at the county spatial scale. Ultimately, we provide an estimate of the relative risk of human encounters with *B. burgdorferi* s.s.-infected nymphs throughout the suitable range of *I. scapularis.* We chose county as our unit of analysis to align with the spatial scale used by the national tick and tick-borne disease surveillance programs [7, 14].

## Methods

### Reported nymphal tick surveillance data used in analyses and validations

For development of the NIP model and validation of estimated NIP and DIN, we used site-level tick surveillance and testing data reported to the CDC’s ArboNET Tick Module (2004–2023). The Tick Module, developed in 2018 with the initiation of the National Tick Surveillance Program [7], houses standardized tick surveillance records from state and public health agencies as well as historical records from CDC-funded tick surveillance studies (e.g., [11, 15, 16]). Reported surveillance data in the Tick Module contain information on the collection site (location, date), collection method (e.g., drag, walking, or CO_2_ sampling), specimens collected (species, life stage, and count collected), and any associated pathogen testing results. If the goal of collection was density sampling, the area sampled (m^2^) was also reported. Pathogen testing results come from tick testing at external laboratories or through the CDC’s Division of Vector-Borne Diseases laboratory in Fort Collins, Colorado. External laboratories were asked to confirm that their testing methods (species-specific molecular assays) adhered to CDC guidance to ensure comparability of reported detections and prevalence estimates [14]. *Ixodes* ticks submitted to CDC were individually tested for five pathogens (*B. burgdorferi* s.s., *Borrelia mayonii*, *Borrelia miyamotoi*, *Anaplasma phagocytophilum*, and *Babesia microti*) using a TaqMan testing algorithm (prior to 2022) [17] or multiplex polymerase chain reaction (PCR) amplicon sequencing (MPAS) assay (2023 onwards) [18]. Summarization of these reported surveillance and testing results can indicate the presence of tick populations and circulation of pathogens as well as life stage-specific densities, infection prevalence, and densities of infected ticks.

### Environmental data used to build NIP model

We considered land cover and bioclimatic conditions aggregated to the county to estimate averaged county-level NIP across the eastern United States. We defined “eastern USA” to encompass states in the contiguous USA that are estimated to be environmentally suitable for *I. scapularis* [19–21]; these states are split by or completely east of the 100th meridian (Additional File 1: Fig. S1). While we included site-level pathogen testing data for fitting the NIP model, we could not include site-level environmental data because 362 (17%) site-years were missing coordinates, and we were uncertain in the precision of coordinates when provided. For example, coordinates could correspond to the county centroid, closest intersection or city, or other jittered location to protect site privacy, and these practices could have also varied by agency and potentially across years; we were confident though that surveillance activities were correctly attributed to the county. Environmental covariates were not temporally and spatially scaled to specific collection events. Instead, they captured long-term average environmental conditions at county scales across the eastern USA, aligning with our goal of describing broad spatial patterns in NIP rather than identifying causal associations and predicting fine-scale spatial and temporal variation in NIP.

We calculated county-level coverage by croplands, forests, and urban areas from the 2019 US Geological survey (USGS) National Land Cover Database [22]. Using the 30-m resolution raster, we calculated the percent of each county covered by croplands (“hay/pasture” and “cultivated crops” categories), forests (“deciduous”, “evergreen”, and “mixed” categories), low-intensity urban areas (“developed-open space” and “developed-low intensity” categories), and moderate-/high-intensity urban areas (“developed-medium intensity” and “developed-high intensity” categories). The developed land cover categories we used to define our urban intensity metrics were based on amounts and types of impervious surfaces (e.g., hard surfaces including rooftops, parking lots, and streets that are impermeable to infiltration of rainfall to underlying soils or groundwater). The low-intensity urban category contained < 50% impervious surfaces (i.e., mixed with vegetation like lawns, golf courses, and parks) while our moderate-/high-intensity urban category contained ≥ 50% impervious surfaces. Forest and low-intensity urban cover likely capture habitats and microclimates suitable for tick and host populations, while croplands and moderate-/high-intensity urban cover likely capture areas largely unsuitable for either tick or host populations.

We calculated county averages of each of the 19 bioclimatic variables using 1000-m resolution rasters from WorldClim [23, 24]. These predefined bioclimatic variables captured long-term trends and seasonality in temperature and precipitation as well as potentially ecologically limiting conditions (e.g., total precipitation during the hottest month of the year, minimum annual temperature). The bioclimatic variables we used were based on 30-year (1970–2000) averages of monthly weather data.

We used hierarchical clustering to choose a set of rather uncorrelated environmental covariates for model fitting (all pairwise Pearson correlations <|0.55|). Hierarchical clustering groups covariates based on patterns of pairwise correlations with other covariates such that covariates in a cluster are highly correlated with each other and have relatively similar patterns of correlation to covariates in other clusters. Using the *klaR* R package [25], we grouped variables from counties with included nymphal infection data into five clusters (Table 1); resulting clusters had relatively high magnitudes of within-cluster correlations and relatively low magnitudes of between-cluster correlations. Within each cluster, we first selected the single covariate with the highest within-cluster correlation to select a single representative covariate from that cluster. If any covariate had a within-cluster correlation < 0.5, we then also selected this covariate. For candidate covariates selected in this manner, we calculated pairwise Pearson correlations and confirmed that all correlations were <|0.55|.

**Table 1:** Environmental variables considered for fitting generalized additive model (GAM) of nymphal infection prevalence

Cluster^a^	Candidate covariate for GAM (county-level)	Avg |within-cluster correlation|	Avg |correlation| to closest cluster	Closest cluster
1	**Percent forest cover**	0.75	0.37	3
	Percent cropland	0.75	0.35	5
2	**Percent open-space and low-intensity urbanization**	0.78	0.23	3
	Percent medium- and high-intensity urbanization	0.78	0.24	1
3	**Temperature seasonality (Bio4, °C)**^b^	0.85	0.40	1
	Temperature annual range (Bio7, °C)^c^	0.82	0.31	1
	Total precipitation of the coldest quarter (Bio19, mm)	0.81	0.49	1
	Total precipitation of the driest quarter (Bio17, mm)	0.81	0.49	1
	Mean temperature of wettest month (Bio11, °C)	0.81	0.53	5
	Min. temperature of coldest month (Bio6, °C)	0.80	0.49	5
	Total precipitation of the driest month (Bio14, mm)	0.80	0.51	1
	Total annual precipitation (Bio12, mm)	0.78	0.53	4
	Precipitation seasonality (Bio15, %)^d^	0.75	0.42	1
	Isothermality (Bio3, %)^e^	0.74	0.51	4
	Mean temperature of driest quarter (Bio9, °C)	0.72	0.42	5
	Annual mean temperature (Bio1, °C)	0.68	0.68	5
4	**Total precipitation of the wettest quarter (Bio16, mm)**	0.72	0.45	3
	Total precipitation of the wettest month (Bio13, mm)	0.69	0.45	3
	Total precipitation of the warmest quarter (Bio18, mm)	0.68	0.29	3
	**Mean diurnal temperature range (Bio2, °C)**^f^	0.37	0.30	1
5	**Max. temperature of warmest month (Bio5, °C)**	0.70	0.38	3
	Mean temperature of warmest quarter (Bio10, °C)	0.69	0.43	3
	**Mean temperature of wettest quarter (Bio8, °C)**	0.41	0.35	1

Set of selected covariates (bold) used for fitting the GAM had pairwise Pearson correlations <|0.55|. A quarter was defined as three consecutive months. Land cover data were calculated from the USGS National Land Cover Database [22] and bioclimatic variables from WorldClim [23]. For more information on derivation of bioclimatic variables, see O’Donnell and Ignizio [63]

^a^Group of variables from hierarchical clustering using 1−|correlation| as the distance measure between pairs of variables. Variables within a cluster have similar patterns of correlation with other candidate covariates

^b^Standard deviation of monthly average temperature, multiplied by 100 to preserve significant digits

^c^Max. temperature of the warmest month (Bio5) − min. temperature of the coldest month (Bio6)

^d^Coefficient of variation of monthly total precipitation, multiplied by 100 to preserve significant digits

^e^[Mean diurnal temperature range (Bio2)/temperature annual range (Bio7)] ×100. Multiplication by 100 for preserving significant digits

^f^Mean of monthly temperature range (monthly max. temperature − monthly min. temperature)

### Estimation of county-level NIP using a GAM

We fitted a binomial GAM [26] using the *mgcv* R package [26, 27] with site-level nymphal *B. burgdorferi* s.s. testing results (prevalence) and smooth functions of county-level environmental covariates. We only included testing results for *I. scapularis* nymphs collected through drag or flag sampling, thus representing *B. burgdorferi* s.s. infection prevalence in nymphs displaying host-seeking behavior that puts humans at risk for tick encounters. We selected a GAM due to its ability to describe nonlinear associations of covariates and to account for variation in NIP attributable to sample size and year of collection. We used thin-plate splines for smooth terms of environmental variables because these are isotropic and have been shown to be the optimal smoother for any given basis dimension [28]. We included an offset for the corresponding number of nymphs tested [i.e., log(number of nymphs)] to account for sample size and a random effect for county using ridge penalties (i.e., equivalent to assuming independent and identically distributed (iid) normal random effects) to account for repeated sampling in counties. We also used weights such that testing results in more recent years contribute to the model’s log likelihood more than those longer ago in time; we numbered years such that 2004, the first year of included surveillance data, was 1. As suggested by Wood [27], we normalized these weights [i.e., collection year/mean (collection years)] to re-weight the contribution of sites without changing the magnitude of the log likelihood.

We used backward selection to select the final covariates in our model. We initially include the seven candidate covariates selected through hierarchical clustering (Table 1), with each environmental covariate fitted as a smooth function using the default basis dimension (*k* = 10). Basis dimension is analogous to the number of knots used to control smoothing. We then iteratively removed non-significant covariates (*P* > 0.05) to reach the final model, comparing Akaike information criterion (AIC) [29] and using ΔAIC > 2 as support for “significant” differences between models. If any covariate’s smooth function had an estimated degrees of freedom (edf) close to 1, indicating a linear relationship, we refitted the model with these covariates as linear terms, and compared the change in AIC. We retained the smooth function if using the parametric version of the covariate led to a reduction in AIC < 2. For these model comparisons, we used the maximum likelihood (ML) estimation method for smoothing parameter estimation. For the final model, we used the restricted estimation maximum likelihood (REML) method for unbiased estimation of variance.

Using the final fitted GAM, we estimated county-level NIP for all counties in the eastern USA. We simulated 1000 random draws from this distribution of the fitted model, a multivariate normal distribution with mean equal to the estimated model coefficients and Bayesian posterior covariance matrix of the parameters that accounted for smoothing parameter uncertainty [27]. We used the mean of these 1000 posterior samples as the county-level prevalence estimate along with the 2.5% and 97.5% quantiles to represent the lower and upper 95% credible interval (CrI) bounds, respectively. For the estimation, we set the random effects for county to zero.

### Evaluation of fitted GAM

We evaluated model fit and performance through a variety of diagnostic metrics. First, we examined standard residual plots (i.e., QQ plot, linear predictor vs. residual scatterplot, histogram of residuals, and fitted vs. observed scatterplot) to assess model fit, using Pearson residuals. Examination of the Pearson residuals allowed further evaluation of model performance by identifying outliers (magnitude > 2) where the model did not fit well to these data (“high” residuals); magnitudes > 4 are considered “extreme” residuals. As we used default basis dimensions in model fitting (*k* = 10) for controlling the “wigglyness” of smooth functions, we assessed whether the basis dimensions (i.e., degree of smoothing) for the smooth terms were appropriate using the method proposed by Wood [27]. This method computes residual variance to identify remaining patterns in residuals through simulations that might indicate that basis dimensions are too small. We also calculated concurvity (range 0–1), a measure of collinearity for smooth functions to confirm that fitted smooths were not confounded with each other.

We calculated Moran’s *I* to assess the spatial autocorrelation of model residuals using the *ape* R package [30]. Due to spatial uncertainty and missing site-level coordinates, we randomly selected points within counties to correspond to each unique testing result using the *sf* R package [31, 32]. For calculating Moran’s *I*, we used inverse distance (Euclidean distance) weighting between points. To capture a range of potential spatial autocorrelations, we create 10 sets of randomly selected points, calculating Moran’s *I* for each set.

### Evaluation of county-level NIP estimates

We evaluated estimated NIP using two separate ArboNET-derived metrics. First, we compared fitted NIP with county-level prevalence estimates derived from site-level testing results with ≥ 25 nymphs tested (prediction error). Next, we compared estimated NIP with reported detection of *B. burgdorferi* s.s. in host-seeking *I. scapularis* ticks of any life stage (validation of predicted spatial patterns of pathogen presence).

While we fitted the GAM with site-level testing results derived from any number of nymphs, we evaluated the fitted estimates against average county-level observed estimates based on testing ≥ 25 nymphs to reduce the impact of small sample sizes on the resulting prevalence estimate. To calculate observed county-level NIP, the estimate routinely derived in tick surveillance, we followed the same method as Foster et al. [33]. Briefly, we averaged estimated prevalence of site-years with ≥ 25 nymphs tested. We then averaged these county-year prevalence values to obtain a single county-level estimate representing the average nymphal prevalence across all sampling locations and years with surveillance for that county. We used mean absolute error (MAE) to quantify prediction error between fitted and observed county-level NIP. We carried out threefold cross-validation 10 times to assess model robustness. We included or excluded all testing data for a county within a fold.

To validate predictions of spatial patterns of pathogen presence, we used receiver operator characteristic (ROC) curve analysis (*pROC* R package [34]) to identify the predicted NIP threshold that maximized the sum of sensitivity and specificity relative to reported detection of *B. burgdorferi* s.s. Using this threshold and the 95% CrI bounds on predicted NIP, we calculated model diagnostic metrics using contingency table analysis. For this, we categorized counties as low (upper bound of 95% CrI below threshold), equivocal (95% CrI contains threshold), and moderate–high (lower bound of 95% CrI above threshold). Note that our use of “low” and “moderate–high” for this categorization were simply for designating relative differences and not necessarily reflective of biologically meaningful differences in magnitudes of NIP. Due to model uncertainty, we could not confidently categorize some counties as low or moderate–high, so we did not include these equivocal counties in the contingency table analysis. We calculated sensitivity as the proportion of counties with reported *B. burgdorferi* s.s. that were categorized as moderate–high and specificity as the proportion of counties with no records of reported *B. burgdorferi* s.s. that were categorized as low. We calculated positive predictive value as the proportion of counties categorized as moderate–high with reported *B. burgdorferi* s.s. and negative predictive value as the proportion of counties categorized as low that had no records of *B. burgdorferi* s.s. circulation.

Considering covariates in the final fitted GAM, we also calculated two extrapolation metrics, Shape and multivariate environmental suitability surface (MESS). These metrics, commonly used in species distribution modeling, can be used to identify covariate values (either singly or in combination) outside the range used in model fitting. Model estimates in dramatically non-analogous conditions may not be robust or biologically meaningful. For this calculation, we compared covariate values in the training set (counties with site-level testing data used to fit the GAM) with those in the rest of the eastern USA (projected set) for which we applied modeled relationships (i.e., “projected” the fitted relationships). The Shape metric represents the multivariate distance in covariate space between each point in the projected set and the closest point in the training set, accounting for dispersion across all points in the training set [35]. Larger values of Shape indicate that conditions are more different in the projected location than those in the training set. MESS uses the Euclidean distances between the center of the training data and the value in the projected location for each covariate independently to identify the single most dissimilar condition for each location in the projection set [36]. We used the *flexsdm* R package to calculate Shape [37] and the *modEvA* R package to calculate MESS [38].

### Estimation of county-level DIN using NIP and DON estimates

To estimate DIN per 1000 m^2^, we multiplied county-level NIP (%) estimated from the GAM by county-level nymphal density (nymphs per 1000 m^2^) previously estimated using a zero-inflated negative binomial model [13]. To incorporate model uncertainties into this estimate, we simulated 1000 random draws using the multivariate normal distribution of each fitted model, resulting in 1000 estimates of DIN per county. Each model’s multivariate normal distribution had a mean equal to the estimated model coefficients and variance using the covariance matrix of the coefficients. We used the median of these 1000 sample estimates as the county-level DIN estimate along with the respective 2.5% and 97.5% quantiles to represent the lower and upper 95% confidence interval (CI) bounds.

### Evaluation of county-level DIN estimates

We evaluated estimated DIN against two separate ArboNET derived metrics. First, we compared estimated DIN with observed county-level DIN derived from field sampling to quantify prediction error. As an alternative evaluation method, we also compared estimated DIN with reported presence of *B. burgdorferi* s.s. circulating in any life stage of *I. scapularis* ticks (i.e., same data as used in the ROC curve-based evaluation of estimated NIP above) and reported collections of any number of host-seeking nymphs. These observations provided additional evidence of enzootic transmission of *B. burgdorferi* s.s. and the presence of host-seeking nymphs, but inclusion criteria were less restricted compared with requirements for estimating DIN.

To calculate county-level observed DIN, we followed the method described recently by Foster et al. [12]. Briefly, we estimated site-level DIN (infected nymphs per 1000 m^2^) by multiplying the maximum DON observed during the peak of nymphal host-seeking period (May–July) by the corresponding site-year NIP. For this, we only included density estimates from collections with ≥ 750 m^2^ dragged or flagged and only infection prevalence for site-years with ≥ 25 nymphs tested. If DON equaled zero for a site (i.e., no nymphs were collected), then we set DIN equal to zero for that site. We then averaged site-level DIN estimates per county and year and selected the maximum county-year estimate. This single county-level DIN captured the highest average DIN (per 1000 m^2^) observed for that county across the years sampled.

### Categorization of DIN and its predictive performance evaluation

We categorized counties into relative risk categories for human encounters with infected nymphs based on their 95% CIs. We use “relative risk” to denote differences in relative magnitudes of DIN with the assumption that similar human behaviors between locations lead to similar encounters with infected nymphs. We identified four potential cut points to dichotomize predicted and observed DIN which varied in their diagnostic performance and spatial distribution on relative risk: 25th percentile of non-zero observed DIN, 90% sensitivity, 95% sensitivity, and the minimum non-zero observed DIN. We chose the 25th percentile of non-zero observed DIN for consistency with previous categorization schemes of acarological metrics [12, 13, 39]. We used 90% and 95% sensitivity thresholds, which resulted in increased geographical area estimated to have elevated DIN, to reflect greater confidence in non-zero DIN estimates relative to zero DIN estimates given the resource-intensive nature of calculating DIN from field observation and inclusion in our calculations. Ecological niche models based on presence-only data have chosen these thresholds using a similar rationale: higher confidence in presence than pseudo-absence points [19, 21]. We also used the minimum observed DIN as a cut point as a conservative approach aimed to capture all potential areas of non-zero DIN.

Using each cut point, we categorized counties as minimal (upper bound of 95% CI below cut point), uncertain (95% CI contains cut point), and elevated (lower bound of 95% CI above cut point) relative DIN. We evaluated diagnostic performance for each cut point using contingency table analysis, including only counties with an estimated DIN of minimal or elevated relative DIN; due to model uncertainty we were not confident in assigning “uncertain” relative DIN counties to either minimal or elevated risk categories, so we excluded these counties from this analysis. Similarly, note that the identification of the 90% and 95% sensitivity cut points was based on counties categorized as only minimal or elevated relative DIN, not uncertain. We calculated sensitivity as the proportion of counties with observed elevated relative DIN that were categorized as elevated relative DIN and specificity as the proportion of counties with observed minimal relative DIN that were categorized as minimal relative DIN. We calculated the positive predictive value as the proportion of counties categorized as elevated with observed elevated relative DIN and negative predictive value as the proportion of counties categorized as minimal with observed minimal relative DIN.

We also performed an external validation of the relative risk categories in each categorization scheme using county-level reported collections in the ArboNET Tick Module of host-seeking nymphs and presence of *B. burgdorferi* s.s. circulating in any life stage of *I. scapularis* ticks (pathogen detection). Considering these components of DIN separately and with fewer constraints than when estimating acarological metrics, we aimed to capture a broader set of counties with evidence of infected host-seeking nymphs with this validation set. We identified collection of host-seeking nymphs as drag or flag sampling that reported ≥ 1 nymph collected; we did not apply date or sampling area constraints to these collections like we did for calculation of DON and DIN. A county could have no collections of host-seeking nymphs because drag or flag sampling resulted in no nymphs collected or no drag or flag sampling occurring. We categorize counties as having neither pathogen nor nymphal collections (“neither”), only pathogen detections (“pathogen”), only nymphal collections (“nymphs”), or both pathogen and nymphal collections reported (“both”). For each relative DIN categorization, we calculated diagnostic metrics derived from contingency tables. For this calculation, we only included counties categorized as minimal or elevated relative DIN as above, as well as counties with neither or both pathogen and host-seeking nymph collections. We calculated sensitivity as the proportion of counties with both reported pathogen circulation and nymphal collections that were categorized as elevated relative DIN and specificity as the proportion of counties with no reports of pathogen circulation or nymphal collections that were categorized as minimal relative DIN. We calculated positive predictive value as the proportion of counties categorized as elevated with both reported pathogen circulation and nymphal collections and negative predictive value as the proportion of counties categorized as minimal with no reports of pathogen circulation or nymphal collections.

### Statistical program details

All analyses and visualizations were performed in R (version 4.4.1) [40]. In addition to those mentioned elsewhere, we used the *cowplot* [41], *dplyr* [42], *ggplot2* [43], and *tidyr* [44] R packages.

## Results

### Overview of tick surveillance data for training the GAM

Testing results for *B. burgdorferi* s.s. in *I. scapularis* nymphs were reported to the ArboNET Tick Module from 2120 sites in 551 counties (30 states); collection records spanned from 2004 through 2023. Averaged county-level prevalence ranged from 0% to 100% (median: 12.0%; Additional File 1: Fig. S2G); high prevalence resulted from testing small numbers of nymphs per site-year.

Across the 551 counties with testing data, surveillance intensity, as measured by the number of nymphs tested and number of sites and years with testing results, varied across counties with nymphal testing data (Additional File 1: Fig. S2). Per county, a median of 11 nymphs (range: 1–506) were tested per site-year, on average (Additional File 1: Fig. S2A), with a median total of 29 nymphs (range: 1–2931) tested across years (Additional File 1: Fig. S2D). Generally, more nymphs were tested in counties in northern areas of the Upper Midwest and Northeast regions. Per county, a median of 1 site (range: 1–10) had testing results per year, on average (Additional File 1: Fig. S2B), with a median total of 2 sites (range: 1–48) with testing results across all years (Additional File 1: Fig. S2E). Similar to counties with larger numbers of nymphs tested, counties with larger numbers of sites were typically in northern areas of the Upper Midwest and Northeast regions. Of the 551 counties, 482 (84.7%) had the most recent testing results from 2018–2023, the period since the initiation of the National Tick Surveillance Program (Additional File 1: Fig. S2C). Of the 69 counties with the most recently reported data prior to 2018, 47 only had reported data from the systematic sampling that informed the previous DIN model (i.e., 2004–2006) [11]. Approximately half of these counties were in the Upper Midwest (*N* = 26), with the remaining in the Northeast (*N* = 12), Southeast (*N* = 7), and Great Plains (*N* = 2) regions. Counties had testing data from a median of 1 year (range: 1–13, Additional File 1: Fig. S2F). Four counties in Minnesota with long-term surveillance sentinel sites (Clearwater, Houston, Morrison, and Pine) [45] had testing data for 9–13 years, while counties in New York, Pennsylvania, Connecticut, and Vermont had the next longest period (4–10 years).

### Overview of tick surveillance data to evaluate modeled NIP

Of these 551 counties with testing data (training dataset) 243 counties (22 states) had ≥ 1 site with ≥ 25 nymphs tested (evaluation dataset), the threshold used by CDC to display prevalence estimates in public-facing maps [14] and the cut-off we used for calculating county-level prevalence from tick surveillance for evaluations. In these counties, average county-level prevalence ranged from 0% to 56.8% (median 20.8%; Additional File 1: Fig. S3A).

Comparing site-level data included in model training (551 counties) and evaluation (243 counties), 86 counties had ≥ 25 nymphs tested in all collections. Of these, 64 counties had testing data from a single site and year; data from these counties were the same for training and evaluation. In the other 157 counties, the training set included a median of 3 (range: 1–27) more sites than the evaluation set (i.e., included sites with < 25 nymphs tested). These additional sites represented a median of 60% (range: 8.3–93.8%) of all sites included per county in the training set (Additional File 1: Fig. S3B) and were included in a median of 75% (range: 16.7–100%) of the 1–13 county-years of nymphal tick testing data per county (Additional File 1: Fig. S2F, S3C). Taken together, this indicates that while a quarter (64/243; 26.3%) of counties included in both the training and evaluation sets had the same data, observed NIP in the majority of counties present in the evaluation set represented a spatial (within county-year) and temporal (across years) average of a subset of the site-level training data, resulting in overlapping but distinctly processed datasets.

**Fig. 1:**
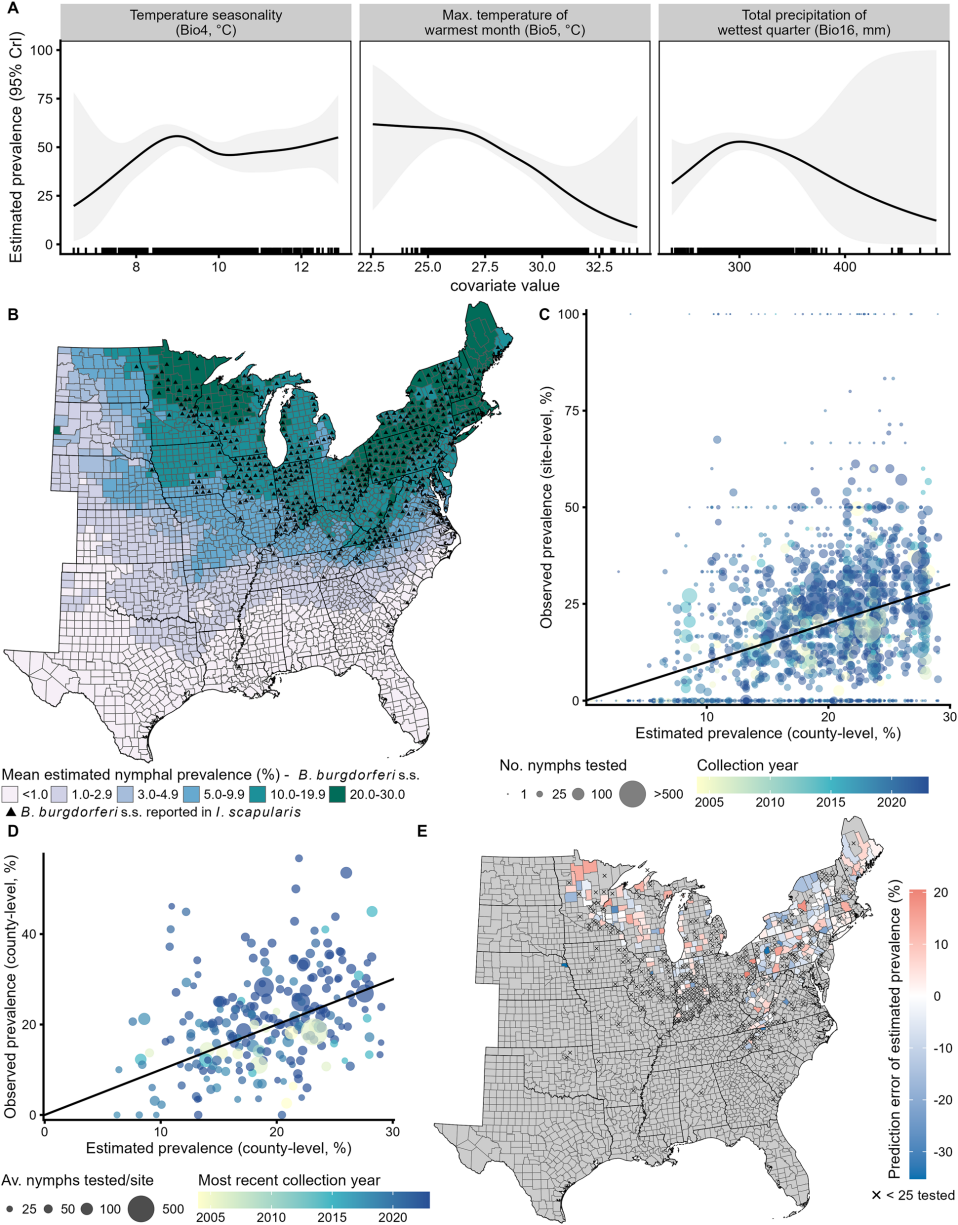
Estimated nymphal infection prevalence with *B. burgdorferi* s.s. (NIP). **A** Fitted smooth functions of bioclimatic covariates in the final generalized additive model (GAM, see Additional File 1: Table S1 for further model details). Shading illustrates the 95% credible interval (CrI). Rug plot illustrates covariate values in the training dataset. For interpretability, covariate values for Bio4 were divided by 100 (see Table 1 for details on derivation). **B** County-level estimated NIP across the eastern USA with reported detection of *B. burgdorferi* s.s. in *I. scapularis* indicated (triangles). **C** Scatterplot of estimated county-level NIP and observed site-level NIP used to fit the GAM. Number of nymphs tested per site (GAM offset) and collection year (GAM weights) indicated by point size and color, respectively. The black diagonal line illustrates the 1:1 relationship. **D** Scatterplot of estimated and observed county-level NIP. The black diagonal line illustrates the 1:1 relationship. Observed NIP calculated using only sites with ≥ 25 nymphs tested (see Fig. 2A for map of observed NIP). Point size represents the average number of nymphs tested per site across years, and color represents the most recent year with testing data associated with that county. **E** Difference in county-level estimated and observed NIP. Positive error (red) indicates that estimated NIP overpredicted observed NIP and negative (blue) indicates underprediction. Counties for which all sites had < 25 nymphs tested are indicated with an X. Gray counties did not have reported nymphal testing data

*Borrelia burgdorferi* s.s. has been detected in any life stage of *I. scapularis* in 511 (19.0%) counties in the eastern USA (Fig. 1B). Nearly all detections (94.1%) occurred in the Upper Midwest (*N* = 255) and Northeast (*N* = 226) regions, with 27 counties in the Southeast and two in the Great Plains also reporting detections.

### Overview of tick surveillance data for evaluating estimated DIN

A total of 484 counties in 34 states had DIN estimates derived from site-level tick surveillance. Of these, 302 counties (62.4%) were assigned a DIN of zero because no nymphs were collected (DON = 0), while four counties had a DIN of zero because no collected nymphs tested positive for *B. burgdorferi* s.s. (NIP = 0). Non-zero DIN from 178 counties in 22 states ranged from 0.11 to 85.26 infected host-seeking nymphs per 1000 m^2^.

We identified 411 counties with both reported collection of host-seeking *I. scapularis* nymphs and detection of *B. burgdorferi* s.s. in *I. scapularis* populations, and 1944 counties with neither pathogen nor host-seeking nymphs reported (Additional File 1: Fig. S4). Of the remaining 339 counties in the eastern USA, 100 had only detection of *B. burgdorferi* s.s. and 239 only had collections of host-seeking nymphs. The majority of counties in the Upper Midwest and Northeast have evidence of both host-seeking nymphs and pathogen circulation, with a mix of counties only reporting one or the other.

### Final fitted GAM

The final GAM included smooth functions of temperature seasonality (Bio4), maximum temperature of the warmest month (Bio5), and total precipitation of the wettest quarter (Bio16) and a random effect smooth function for county, with all smooths statistically significant (Bio16 smooth *P* = 0.002, all other smooths *P* < 0.001, Additional File 1: Table S1). All estimated concurvity < 0.35 indicated that the three bioclimatic smooths were not confounded with each other.

The fitted smooth functions (Fig. 1A) showed support for a positive association between NIP and temperature seasonality (Bio4), a negative association with maximum temperature of the warmest month (Bio5), and a unimodal association with total precipitation of the wettest month (Bio16). Prevalence was predicted to increase with increasing temperature seasonality up to about 9 °C (i.e., 9 °C standard deviation in monthly temperatures) and remain relatively flat afterwards. As total precipitation of the wettest quarter increased, prevalence was expected to increase, up to about 300 mm, and then decrease thereafter. Unsurprisingly, covariate values with few observations in the training dataset had higher uncertainty (i.e., wider CrIs). Of note, the 95% CrI spanned nearly 0–100% predicted prevalence with > 450 mm total precipitation in the wettest quarter (Bio16); five sites had values of Bio16 above this threshold and were all located in the Southeastern USA (Georgia, North Carolina, and South Carolina). See Additional File 1: Table S2 for ranges of covariates in the training set and across the eastern USA.

### Evaluation of fitted GAM

Examination of residual diagnostic plots for the GAM did not indicate any major problems with the final fitted model (Additional File 1: Fig. S5). The scatterplot of response versus fitted values exhibited horizontal lines corresponding to prevalence estimates from small sample sizes (i.e., 0%, 33%, 50%, and 100%). The QQ plot and histogram suggested that the residuals were leptokurtic (light-tailed). Counties with testing results from a single site had small residuals (near zero; Additional File 1: Fig. S6), likely contributing to the leptokurtic nature of the residuals. However, small residuals were not unique to counties with few sites (Additional File 1: Fig. S7).

Of the 2120 Pearson residuals, 165 (7.8%) had a magnitude > 2 (“high” residual). Of these, five had a magnitude > 4 (“extreme” residuals). These residuals occurred in sites in Connecticut (Fairfield Co., 2023), North Carolina (Mecklenburg Co., 2020), New York (Onondaga and Suffolk counties, 2023), and Pennsylvania (Fayette Co., 2023). All “extreme” residuals were positive, indicating that the model underpredicted these site-level estimates (observed NIP: 50–100%; Additional File 1: Fig. S7). In contrast, “high” residuals with magnitudes < 4 had both negative and positive signs. Negative-signed “high” residuals (overprediction) occurred in some instances with site-level prevalence estimated < 22.0%, while positive-signed “high” residuals (underprediction) only occurred in instances with site-level prevalence > 18.2%. While all “extreme” residuals occurred in more recent collections, we did not identify environmental conditions, collection attributes, or surveillance intensity metrics uniquely associated with only “high” or “extreme” residuals (Additional File 1: Fig. S7), supporting a robust model performance. The largest magnitudes of site-level residuals occurred primarily in the Northeast, with large magnitudes also present in the Upper Midwest (Additional File 1: Fig. S8). While there was heterogeneity across counties, average county-level residuals tended to be lower (more negative) in the Upper Midwest than the Northeast.

Of our 10 sets of randomly placed points, eight indicated the presence of positive spatial autocorrelation among residuals (Moran’s *I* *P*-value < 0.05; see Additional File 1: Table S3), indicating that some spatial patterns were not fully captured by the GAM.

### Estimates of county-level NIP from fitted GAM

**Fig. 2:**
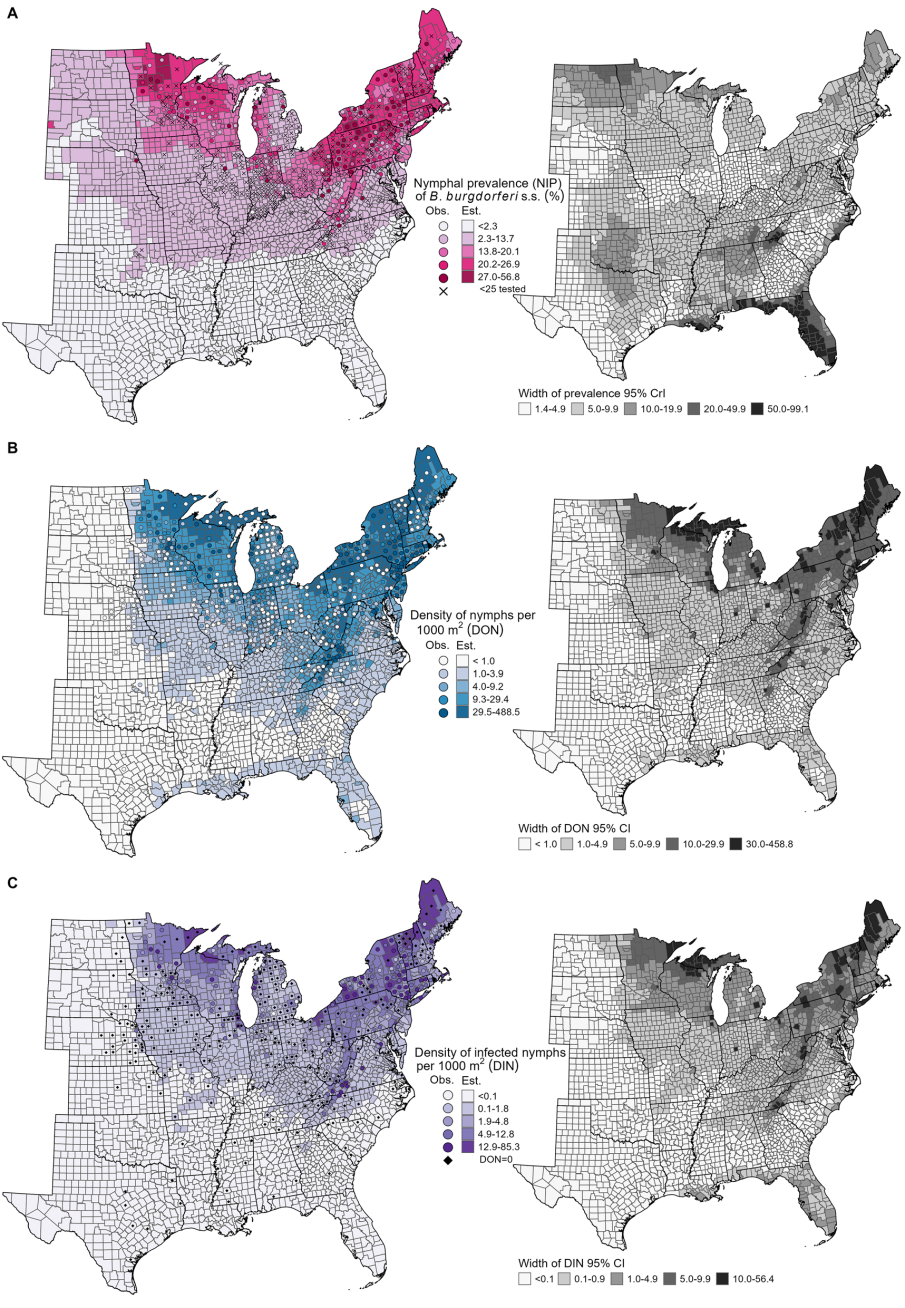
Estimated and observed acarological metrics (left) with their corresponding uncertainty  (right). We multiplied county-level modeled **A** nymphal infection prevalence of *B. burgdorferi* s.s. (NIP) with **B** density of host-seeking nymphs (DON) to estimate the **C** density of host-seeking *B. burgdorferi* s.s.-infected nymphs (DIN). NIP was estimated with a generalized additive model and DON with a previously described zero-inflated negative binomial model [13]. Uncertainty presented as the width of the 95% credible interval (CrI; NIP) or 95% confidence interval (CI; DON and DIN). Categories for shading counties (estimated) and centroids (observed) based on observed values of that metric derived from tick surveillance; “zero” (i.e., below the minimum observed value) and quartiles of the non-zero observed metric. Counties with all testing results for < 25 nymphs indicated with an X in **A**). Counties for which we assigned DIN = 0 because no host-seeking nymphs were collected (DON = 0) are indicated with a black diamond in **C**. Est: estimated; Obs: observed

Geographically, the GAM predicted higher NIP (20–30%) in northern areas in the Upper Midwest and Northeast regions and lower NIP (< 1%) throughout the Southeastern USA (Figs. 1B, 2A). Somewhat higher NIP (3–10%) was estimated in northern parts of the Great Plains states. Temperature seasonality (Bio4, Additional File 1: Fig. S9A) and maximum temperature of the warmest month (Bio5, Additional File 1: Fig. S9B) largely described the north–south division in NIP. High temperature seasonality (Bio4, standard deviation in monthly temperature > 9 °C) and low maximum temperature of the warmest month (Bio5, < 28 °C) characterized counties in the Northeast and Upper Midwest regions with the highest estimated NIP. Precipitation of the wettest quarter (Bio16, Additional File 1: Fig. S9C) described east–west variation, with low precipitation amounts (< 200 mm) associated with low estimated NIP across the Great Plains. A highly forested county (Lawrence County, South Dakota) was estimated to have a high NIP (21%) as it was cooler and wetter than surrounding primarily prairie counties (Additional File 1: Fig. S9).

County-level prevalence estimates from the GAM showed a positive relationship with the site-level estimates used for model fitting (Fig. 1C). The largest deviations between site- and county-level estimates occurred for sites with few nymphs tested. For example, in counties with model estimates of 10–30%, site-level estimates of 50% or 100% resulted from a single nymph testing positive out of two or one tested, respectively. Collection year appeared to contribute to site-level scatter around modeled NIP, without a systematic bias in direction of deviation. As such, fitted county-level NIP smoothed over site-level and year-to-year heterogeneity. The fitted GAM had fair deviance explained (39.1%), indicating good model fit in the context of this site-level variation.

Uncertainty, as measured by the width of the 95% CrI of NIP, varied across the eastern USA (Fig. 2A). The widest CIrs were in counties along the Gulf Coast and at the southern tip of the Appalachian Mountains, in counties with large amounts (> 450 mm) of total precipitation during the wettest quarter (Bio16; Additional File 1: Fig. S9). The fitted smooth for Bio16 had wide CrIs on this upper end of values (Fig. 1A), thus contributing to the wide CrIs for these counties. Counties in the northern parts of the Great Plains and Upper Midwest regions with higher estimated NIP also had higher uncertainty (wider CrIs).

### Evaluation of county-level NIP estimates

Model-estimated NIP exhibited a linear relationship with field-derived estimates of NIP based on sites with at least 25 nymphs tested (Fig. 1D). On average, estimated NIP was different from observed NIP by 7.6% (i.e., MAE = 7.6%). The narrow range in MAE (7.8–8.0%) across testing folds of the cross-validation indicated the model estimates were not overly sensitive to data from any particular county. Observed NIP in counties with larger average numbers of nymphs tested per site (e.g., > 100 tested) were often more similar to estimated NIP than those with smaller numbers tested (e.g., 25–50), but counties with smaller numbers tested also aligned closely with estimated NIP. The model generally overestimated observed NIP in counties where the only prevalence estimates available were derived from the systematic sampling in 2004–2006 [11]. Estimated NIP often underestimated observed prevalence across the Northeast and overestimated observed prevalence in the Upper Midwest, but with county-to-county variation in the direction and magnitude of prediction error (Fig. 1E), which reflected county-to-county variation in observed NIP (Fig. 2A). The model also underestimated NIP in Thurston County, Nebraska (observed NIP: 46.2%; estimated NIP: 10.9%), a county geographically distant from others with testing data and one of only three counties in the Great Plains region with any reported testing data.

The county-level spatial patterns of estimated NIP aligned with reported circulation of *B. burgdorferi* s.s. in *I. scapularis* (Fig. 1B). The area under the curve (AUC) from the ROC curve analysis (0.88) indicated excellent discriminatory power [46]. The majority of counties with reported pathogen circulation in any life stage of *I. scapularis* were located in the Upper Midwest and Northeast regions, areas with the highest estimated NIP. A few counties in the Southeast (one in Louisiana, two in Georgia, and two in South Carolina) with estimated NIP < 1.0% also had reported pathogen circulation, suggesting that while *B. burgdorferi* s.s. is present in *I. scapularis* populations in this area, pathogen detection is not common in host-seeking nymphs. Nymphs in this area quest at or below leaf litter [47], thus resulting in rare human encounters with infected nymphs.

Using the threshold identified by the ROC curve analysis (8.16%) and the 95% CrI of predicted NIP, we categorized counties as low, equivocal, and moderate–high to calculate diagnostic metrics (Additional File 1: Fig. S10). A total of 1628 counties were categorized as low (*N* = 780) or moderate–high (*N* = 848), and the remaining 1066 were categorized as equivocal (i.e., 95% CrI for NIP contained 8.16% threshold). The relatively large number of counties categorized as equivocal derived from the wide widths of the credible intervals of NIP across the eastern USA (Fig. 2A). Counties categorized as moderate–high clustered in the Upper Midwest and Northeast, while counties categorized as low were located along the western edge of the Great Plains and throughout the central Southeastern regions. Of the counties classified as equivocal, 983 had no records of *B. burgdorferi* s.s. in *I. scapularis* ticks, while 83 did report detecting this pathogen. These equivocal counties separated those classified as low or moderate–high as well as along the Gulf Coast from Louisiana throughout Florida. Considering only counties categorized as low or moderate–high, the model correctly categorized 766 counties with no records of pathogen detection as low NIP and 414 counties with reported pathogen circulation as moderate–high NIP (72.5% accuracy). The model correctly identified 96.7% (414/428) of counties with pathogen detection as moderate–high (i.e., sensitivity) and 63.8% (766/1200) of counties without pathogen detected as low (i.e., specificity). The majority of misclassification occurred in southern portions of the Upper Midwest and Northeast as well as Maine, where the model predicted moderate–high NIP but there were no records of *B. burgdorferi* s.s. circulating. Of the 848 counties classified as moderate–high, 414 (48.8%) had reported pathogen circulation (i.e., positive predictive value), and of the 780 counties classified as low, 766 (98.2%) had no records of pathogen detection (i.e., negative predictive value).

**Fig. 3:**
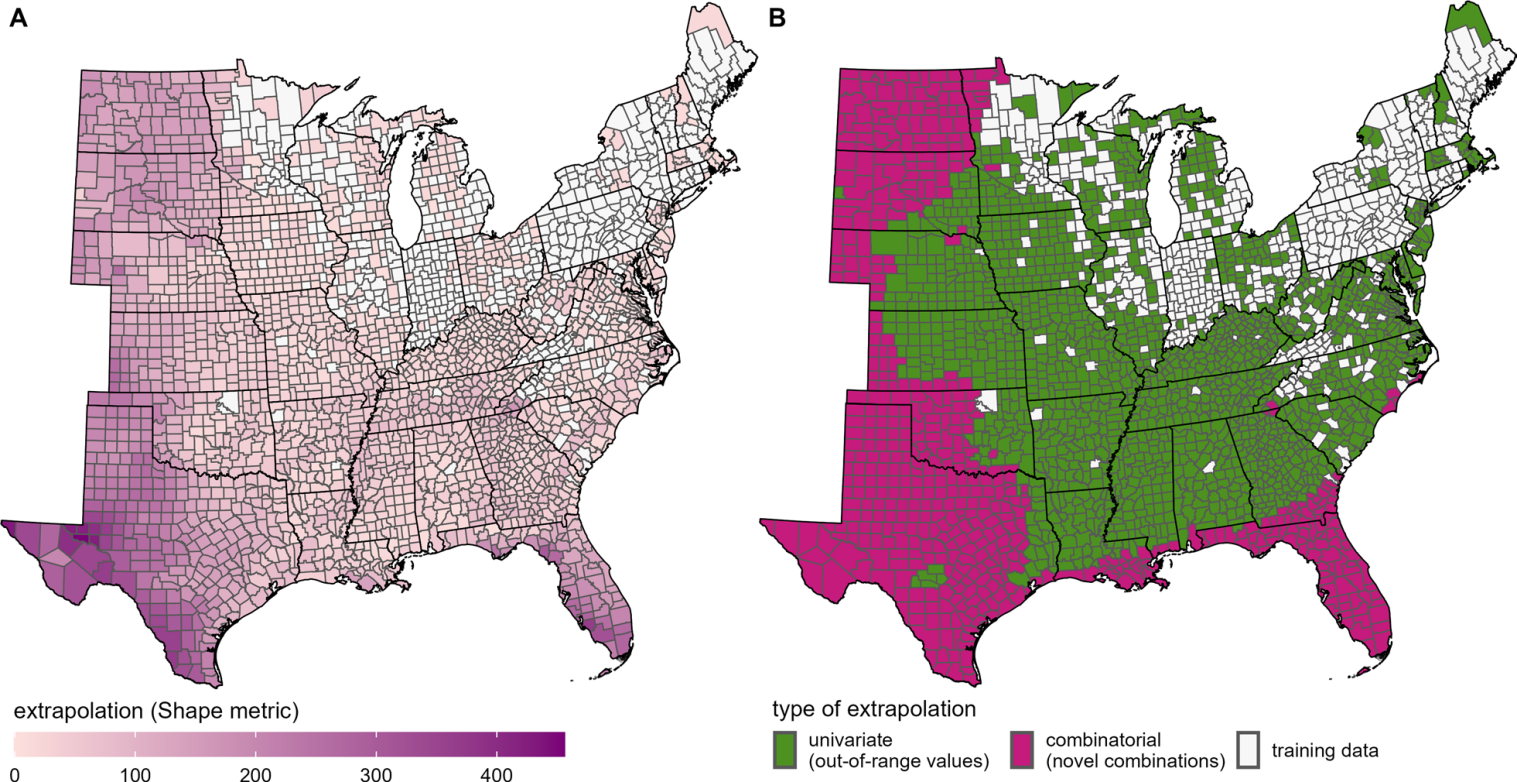
Extrapolation (dissimilarity) between environmental conditions in training and projected sets. Counties used to fit the generalized additive model (training data) and the other counties in the eastern USA to which the fitted model was projected (projected data). Shape used as metric of the **A** magnitude and **B** type of extrapolation. Larger values of Shape indicate more dissimilar environmental conditions between the training and projected datasets. See Additional File 1: Fig. S9 for univariate similarity for each covariate and the multivariate environmental suitability surface (MESS) plot

Unsurprisingly, dissimilarity in environmental conditions increased with increasing distance from counties used to train the GAM (Fig. 3, Additional File 1: Fig. S9). The Shape metric identified novel combinations of environmental conditions occurring throughout the Great Plains states and along the Gulf Coast (Fig. 3B), therefore reducing our certainty in the robustness of estimated NIP in these areas. Counties in the Great Plains experienced lower precipitation amounts during the wettest quarter, while lower temperature seasonality and higher precipitation during the wettest quarter occurred along the Gulf Coast (Additional File 1: Fig. S9). Southwestern Texas experienced the most unique environmental conditions (largest value of Shape, Fig. 3A), with higher maximum temperatures of the warmest month and less precipitation of the wettest quarter compared with other regions (Additional File 1: Fig. S9). Most areas identified as having novel combinations of conditions also had high model uncertainty (wider 95% CrI widths) in estimated NIP (Fig. 2A), except western Texas, Kansas, and Nebraska, which had relatively low CrI widths.

### Estimation and evaluation of county-level DIN

Estimated DIN, calculated by multiplying modeled county-level estimates of NIP and DON [13], defined two distinct areas in the eastern USA. Counties throughout the Great Plains and Southeast were estimated to have low DIN (< 0.11 infected nymphs per 1000 m^2^) while those in the Upper Midwest and Northeast had higher DIN estimates (> 0.11 per 1000 m^2^; Fig. 2C); counties in the northern reaches of the Upper Midwest and Northeast had estimated DIN ranging from 13.1 to 25.6 infected nymphs per 1000 m^2^. Surprisingly, the highest DIN (30.6–42.2 infected nymphs per 1000 m^2^) were estimated in counties in northeastern Ohio, areas predicted to be highly suitable, but with few questing nymphs collected. Low estimated DON (< 1.0 nymphs per 1000 m^2^) along the Great Plains resulted in low DIN in this region. Similarly, low estimated NIP (< 2.3%) and DON (< 1.0 nymphs per 1000 m^2^) throughout the Southeast resulted in low DIN.

High observed DIN were broadly located across the Upper Midwest and Northeast, with the highest observed (85.3 infected nymphs per 1000 m^2^) in New York as well as along the southern end of the Appalachian Mountains in Virginia (58.6 infected nymphs per 1000 m^2^) and North Carolina (54.3 infected nymphs per 1000 m^2^). Estimated DIN underestimated observed DIN in some counties in Virginia, New York, central Minnesota, and Wisconsin (Additional File 1: Fig. S11B) and overestimated observed DIN in some counties in northern Minnesota, Michigan, Maine, Vermont, and New Hampshire where no nymphs were collected (i.e., estimated non-zero DIN with a DON of zero observed, Fig. 2C). These spatial patterns in prediction error for DIN were similar to those for DON (Additional File 1: Fig. S11A); overestimation was noted in locations like Pennsylvania, Ohio, and Vermont, which were more recently colonized by *I. scapularis* populations [5, 13, 48]. In contrast, prediction error in NIP exhibited more variability in magnitude and direction county-to-county (Fig. 1E).

Uncertainty in estimated DIN, as measured by the width of the 95% CI, was highest in counties with higher predicted magnitudes (Fig. 2C). More uncertainty (wider 95% CI) was also estimated in counties along the Gulf Coast and up the Atlantic coast. The Great Plains and majority of the Southeastern regions had narrow confidence intervals and thus high certainty in low estimates of DIN. These patterns mirrored those of estimated DON (Fig. 2B). As expected with a negative binomial model, higher standard errors and larger 95% CI were estimated in areas with higher estimated DON [13]. The relatively large width of the NIP 95% CrI along the Gulf Coast and southern end of the Appalachians in North Carolina likely contributed to the wider confidence intervals for estimated DIN in these areas.

### Categorization of DIN and predictive performance evaluation

**Fig. 4:**
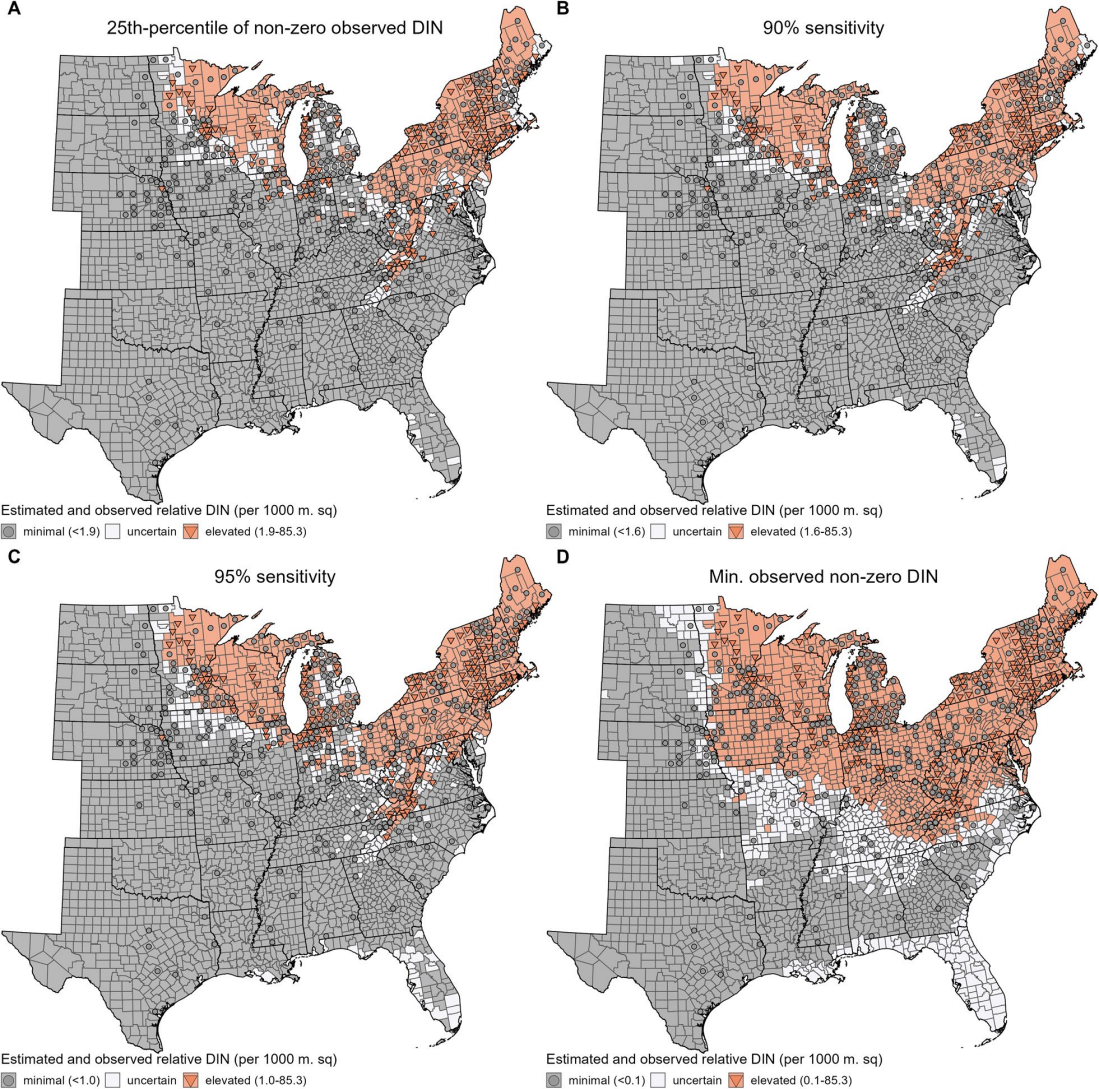
Estimated and observed relative DIN categories. Categorization using 95% CI of estimated DIN and four candidate thresholds: **A** 25th percentile of non-zero observed DIN (1.93/1000 m^2^), **B** cut point resulting in 90% sensitivity (1.60/1000 m^2^), **C** cut point resulting in 95% sensitivity (1.03/1000 m^2^), and **D** minimum observed non-zero DIN (0.11/1000 m^2^). Observed relative DIN based on county DIN estimates calculated from tick surveillance data. Minimal relative DIN classified when the upper DIN 95% CI bound (gray shading) or observed DIN (gray circle) < cut point. Uncertain relative DIN (white shading) classified when the DIN 95% CI contained cut point. Elevated relative DIN classified when the lower DIN 95% CI bound (coral shading) or observed DIN (coral triangle) ≥ cut point. See Additional File 1: Table S4 for calculation of diagnostic metrics for each categorization

We collapsed estimated DIN into broad categories of minimal and elevated relative DIN using four candidate cut points to explore the diagnostic performance and spatial distribution of resulting categories (Fig. 4). Using the 25th percentile of non-zero observed DIN (1.93 infected nymphs per 1000 m^2^) as the cut point resulted in two regions of elevated relative DIN, the northern part of the Upper Midwest (Minnesota, Wisconsin, and Michigan) and the Northeast south along the Appalachian Mountains into North Carolina (Fig. 4A). The remainder of the eastern USA was classified as minimal relative DIN apart from a loose, scattered band of counties along the edge of the elevated regions and in western Florida (*N* = 265) categorized as uncertain (i.e., 95% CI contained cut point). This categorization tended to underestimate observed relative DIN in the southern parts of the Upper Midwest (e.g., Illinois and Indiana) and overestimate observed relative DIN in the Northeast where no nymphs were collected (e.g., Pennsylvania and Maine, Fig. 2C).

We then identified cut points resulting in 90% and 95% sensitivity, reducing the threshold to 1.60 and 1.03 infected nymphs per 1000 m^2^, respectively. This expanded the geographical area of counties categorized as elevated, increasing the number of counties in Iowa, Illinois, Indiana, Ohio, Michigan, and West Virginia, but largely maintaining separation between the two foci of elevated relative DIN (Figs. 4B, C). The number of counties categorized as uncertain remained similar (*N* = 258 for 90% sensitivity cut-off, *N* = 301 for 95% sensitivity cut-off), with an increase in the number of counties categorized as uncertain along the Gulf Coast. These categorizations had similar spatial patterns in misclassification, with somewhat fewer observed elevated relative DIN underestimated in the southern parts of the Upper Midwest (e.g., Illinois and Indiana).

Using the minimum observed DIN (0.11 infected nymphs per 1000 m^2^) to delineate non-zero DIN as the fourth cut point resulted in a single, continuous area of elevated risk encompassing most states in the Upper Midwest and Northeast (Fig. 4D). A much wider band of counties classified as uncertain surrounded this elevated area. Counties along the Gulf Coast, Florida, and up the Atlantic coast were also categorized as uncertain given the large 95% CI estimated here (Fig. 2). A total of 541 counties were categorized as uncertain. This categorization nearly always overpredicted DIN relative to observed DIN (Fig. 4D). All but one county with an observed elevated DIN was classified as having an elevated relative DIN, while counties throughout the elevated region were overpredicted. As above, overestimation mainly occurred in counties where no nymphs were collected.

Using only counties categorized as minimal or elevated relative DIN, as we decreased the cut point, the accuracy, specificity, and positive predictive values decreased while sensitivity and negative predictive values increased (Table 2, Additional File 1: Table S4). The very high negative predictive values (91.1–100%) illustrate that these categorizations did well at capturing areas of minimal risk for encountering infected host-seeking nymphs. In contrast, the relatively poor positive predictive values (45.5–44.5%) suggest that perhaps DIN has not yet reached the peak predicted in climatically suitable locations in the Upper Midwest and Northeast. Alternatively, the model could be overpredicting in these areas. Relatively high sensitivity (84.1–100%) with fair to low specificity (61.8–16.6%) indicates that these categorizations tended to err on the side of overprediction, especially when using the minimum observed non-zero DIN as the cut point.

**Table 2:** Diagnostic metrics (%) of categorization methods for estimated DIN

Cut point^a^	Validation data^b^	Accuracy	Sensitivity	Specificity	PPV^c^	NPV^d^
25th-%tile of non-zero observed DIN (1.93/1000 m^2^)	Observed DIN	67.9 [265/390]	84.1 [90/107]	61.8 [175/283]	45.5 [90/198]	91.1 [175/192]
	Evidence of host-seeking infected nymphs	92.2 [1987/2154]	67.8 [221/326]	96.6 [1766/1828]	78.1 [221/283]	94.4 [1766/1871]
90% sensitivity (1.60/1000 m^2^)	Observed DIN	65.8 [263/400]	89.7 [105/117]	55.8 [158/283]	45.7 [105/230]	92.9 [158/170]
	Evidence of host-seeking infected nymphs	92.0 [1991/2165]	73.5 [258/351]	95.5 [1733/1814]	76.1 [258/339]	94.9 [1733/1826]
95% sensitivity (1.03/1000 m^2^)	Observed DIN	61.5 [248/403]	94.8 [128/135]	44.8 [120/268]	46.4 [128/276]	94.5 [120/127]
	Evidence of host-seeking infected nymphs	90.8 [1935/2132]	82.0 [300/366]	92.6 [1635/1766]	69.6 [300/431]	96.1 [1635/1701]
Min. observed non-zero DIN (0.11/1000 m^2^)	Observed DIN	50.0 [221/442]	100 [177/177]	16.6 [44/265]	44.5 [177/398]	100 [44/44]
	Evidence of host-seeking infected nymphs	76.6 [1419/1853]	99.8 [402/403]	70.1 [1017/1450]	48.1 [402/835]	99.9 [1017/1018]

Numbers in brackets indicate the calculation for each diagnostic metric. See Additional File 1: Tables S3–S4 for associated contingency tables for further details of the categorization and calculations

^a^Cut point (no. infected host-seeking nymphs per 1000 m^2^) used to dichotomize estimated DIN. Observed DIN also dichotomized using the indicated cut point when used as the validation set

^b^County-level data reported to the ArboNET Tick Module used to evaluate diagnostic metrics of relative DIN categorizations. Observed DIN calculated from tick surveillance. Evidence of host-seeking infected nymphs based on reported collections of any nymphs through drag/flag sampling and detection of *B. burgdorferi* s.s. in any life stage of *I. scapularis* ticks

^c^Positive predictive value

^d^Negative predictive value

To further investigate the concordance of the four relative DIN categorization schemes with observed tick surveillance, we compared these categories to a wider, independent set of tick surveillance data including reported detection of *B. burgdorferi* s.s. in *I. scapularis* and collection of host-seeking *I. scapularis* nymphs (Additional File 1: Fig. S4). Notably, 74 of the 302 counties (24.5%) we classified as zero DIN because of a zero DON (Fig. 2C) had evidence of both host-seeking nymphs and pathogen circulation in the wider dataset (Additional File 1: Fig. S12). The majority of these instances occurred in the Upper Midwest (35/156 counties with zero DON with evidence of both) and Northeast (36/68 counties with zero DON with evidence of both) regions. Counties in the Great Plains and Southeast generally had no records of either host-seeking nymphs or pathogen circulation.

Using only counties categorized as minimal or elevated relative DIN and those that have reported neither or both collection of nymphs and pathogen circulation, we calculated diagnostic metrics for the four categorization methods (Table 2, Additional File 1: Table S5). Overall, categorizations based on the three highest cut-offs (1.93–1.03 infected nymphs per 1000 m^2^) had relatively good diagnostic metrics: very high accuracy (92.2–90.8%), moderate sensitivity (67.8–82.0%), very high specificity (96.6–92.6%), fair positive predictive value (78.1–69.6%), and high negative predictive value (94.4–96.1%). These metrics suggest that these categorizations did well capturing areas of true minimal risk (absence of host-seeking nymphs and pathogen; high specificity and negative predictive value) while the elevated categorization somewhat overpredicted risk (fair positive predictive value). The elevated categorizations resulting from these three higher cut-offs spatially aligned with counties reporting both collection of nymphs and pathogen circulation, but missed such counties in Illinois, Indiana, Virginia, and North Carolina (moderate sensitivity). Counties categorized as elevated when using the minimum observed non-zero DIN as the cut-off contained nearly all counties with reported collection of nymphs and pathogen detection, resulting in very high sensitivity (99.8%). However, this resulted in rather poor positive predictive value (48.1%), as the model overpredicted risk, especially in areas of Iowa, Kentucky, and Maine where we have little to no evidence of host-seeking nymphs or pathogen circulation in ArboNET records. With this overprediction, the categorization had fair specificity (70.1%). On the other hand, negative predictive value was excellent (99.9%), indicating that it did very well at capturing areas of true minimal risk.

## Discussion

Since becoming a nationally notifiable condition in 1991, cases of Lyme disease have been reported over an expanding geographical area. High-incidence counties were initially clustered in northeastern coastal communities and in western Wisconsin and eastern Minnesota in the Upper Midwest [49]. Now, counties reporting high incidence of Lyme disease are widely distributed across the Northeast and Upper Midwest regions and into the Appalachian Mountains of Virginia and North Carolina [2–4]. These changes are attributable, in part, to expansion in the geographical ranges of *I. scapularis* and *B. burgdorferi* s.s. [5]. A previous study described systematic collection of DIN data from 2004–2006 and presented an acarological risk model for Lyme disease [11]. Recent tick surveillance activities have shown an increase in acarological risk (DIN) over the past two decades, highlighting a need to update the original DIN model. However, methodological differences in collection of DIN data necessitated a novel approach to derive DIN estimates. The original model was based on a research study that set out to collect ticks over an evenly spaced grid across the eastern USA [11, 50]. Subsequent tick surveillance efforts were opportunistic, with sites selected to meet local public health needs [51], resulting in an uneven and relatively sparse distribution of recent DIN records [12]. To overcome limited recent DIN sampling, we used an alternative modeling approach. Rather than modeling acarological risk using direct field observations of DIN following Diuk-Wasser et al. [11], we optimized the data available in ArboNET to first develop a DON model [13], then developed a NIP model using *B. burgdorferi* s.s. prevalence in host-seeking nymphs. We then combined the resulting modeled estimates to map predicted DIN. Field observations of DIN, coupled with ArboNET records of counties where host-seeking nymphs were collected and where *B. burgdorferi* s.s. was detected in ticks, were used to evaluate performance of the DIN model. Our resulting acarological risk model revealed a substantially wider area over which DIN is expected to be elevated compared with the previous model based on data collected nearly two decades earlier [11].

Our results provide an updated estimate of the spatial distribution and average peak density of infected nymphs across the eastern USA, the estimated climatically suitable range of *I. scapularis* [20, 21]. Previous model-based estimates of DIN [11] used site-year estimates derived from systematic sampling: average daily DON multiplied by site-year NIP. Because the method for calculating DIN differed between our study and the previous model [11], the values were not directly comparable; however, we can compare relative magnitudes of and spatial patterns in estimates. Our model predicted a broader area of high DIN that expands beyond the two foci identified by Diuk-Wasser et al. [11] in the Upper Midwest and Northeast. We estimated a single continuous area of suitable habitat to support high DIN across northern states in the eastern USA. Specifically, we predicted high DIN throughout New York, Pennsylvania, Ohio, northern Indiana, northern Illinois, and Michigan as well as along the Appalachian Mountains south through Virginia and North Carolina. Of note, this expansion in high DIN contained locations previously observed as high but predicted as low DIN (e.g., Michigan and western Pennsylvania and New York) [11]. Similarly, some of our surveillance-derived estimates of DIN calculated from tick surveillance throughout this area of higher estimated DIN were assigned zero values as no nymphs were collected, suggesting overprediction. However, nearly one-third (71/224) of these instances in the Northeast and Upper Midwest had evidence of both the collection of host-seeking *I. scapularis* nymphs and circulation of *B. burgdorferi* s.s., supporting the accuracy of non-zero DIN estimates. Due to the resource-intensive requirements for calculating this metric, observed DIN may underestimate the true risk of encountering an infected host-seeking nymph; too few or no nymphs could be collected and tested to be included in DIN estimation. Further tick surveillance could be warranted to monitor DIN in these previously underestimated areas. Notably, in contrast to Diuk-Wasser et al. [11], our model identified suitable habitat to support DIN in western Virginia and North Carolina, where high DIN and recent Lyme disease cases have been reported subsequent to the data collection used to train the previous DIN model [2, 11, 12].

While we developed the NIP model as an intermediate step in estimating DIN, our county estimates of average prevalence of *B. burgdorferi* s.s. in host-seeking nymphs provides another tool for public health. Relative magnitudes of NIP could contextualize estimated risk of human infections with this tick-borne pathogen and highlight areas of uncertainty where future surveillance efforts might be focused. We recognize a high degree of variability in NIP across small distances and among years within a single sampling location [33, 45] that is not represented in our averaged estimates. Investigation of Pearson residuals indicated the presence of unaccounted spatial patterns. Thus, inclusion of spatial structure could improve future model estimates of NIP. We also note that our method for randomly identifying the spatial location of residuals did not account for time (year of testing), and thus the results from Moran’s *I* may not fully capture the degree of spatial autocorrelation remaining. As such, we caution that our NIP estimates should be used as a guide for identifying regions where *B. burgdorferi* s.s. could be circulating enzootically, rather than using it as a tool for precisely estimating local prevalence. Notably, our model estimates aligned spatially with reported circulation of *B. burgdorferi* s.s. and showed good discrimination between our low and moderate–high prevalence categories. Our NIP map might be useful for making location-based estimates of the relative likelihood that a nymph is infected with *B. burgdorferi* s.s. following recognition of a tick bite. However, as Diuk-Wasser et al. [11] noted, the lack of precision in NIP estimates (observed and predicted) calls into question the validity of relying on exact prevalence estimates or thresholds to guide clinical decisions on treatment.

Previous work has summarized spatial distribution of *B. burgdorferi* s.s. in *Ixodes* ticks [52] and estimated environmentally suitable regions for its circulation [19]. Numerous studies have summarized NIP across sites or years at local or state scales (e.g., [16, 45, 53, 54]) or across regional scales (e.g., [33, 55, 56]). Our results add to this body of knowledge by estimating a smoothed average magnitude of nymphal prevalence of *B. burgdorferi* s.s. across all counties in the eastern USA based on environmental associations. We note that these associations are not causal or predictive of stable conditions but describe the large-scale spatial patterns in observed NIP. We estimated the highest NIP in the Upper Midwest and Northeast regions, with higher prevalence (20–30%) in more northern counties in these areas, and low NIP (< 1%) across the southern areas of the Great Plains and Southeast. These broad spatial patterns align with previously summarized trends [33]. Our results cannot indicate whether NIP in the recently colonized Great Plains will stabilize at magnitudes similar to those in long-established areas or at lower values due to environmental and ecological differences. Relatively large credible intervals and heterogeneity in prediction error compared to observed county- and site-level NIP highlight that these estimates should not be taken as precise magnitudes of NIP. Counties classified as equivocal from the ROC curve analysis highlight areas where, due to model uncertainty, we were unable to classify NIP. These areas could be prioritized for further surveillance to elucidate risk.

Compared to counties with previously reported detection of *B. burgdorferi* s.s. in any life stage of *I. scapularis* [19, 52]*,* our estimates suggest that environmental conditions are suitable to support enzootic transmission of *B. burgdorferi* s.s. further west into the Great Plains states and south into Missouri. However, our GAM model only utilized county-level, long-term average environmental conditions and thus did not include factors like presence of host or tick populations necessary for maintenance of pathogen circulation. Therefore, these estimates could be overestimations, or they may represent areas where enzootic transmission might become established in the future. Tick surveillance records were extremely limited from that region, contributing to uncertainty in our estimates. We emphasize that this is treated as model error because *B. burgdorferi*-infected nymphs have not been widely reported to ArboNET from these regions. A single county in Nebraska (Thurston County) has reported ongoing transmission of *B. burgdorferi* s.s. [57], but it is unclear whether transmission cycles could be established further west as *I. scapularis* tick populations expand. Additional surveillance in these areas could further elucidate the geographical distribution of *B. burgdorferi* s.s. in host-seeking *I. scapularis* ticks and potential risk of human exposure to tick-borne pathogens.

Our relative DIN categorizations delineated areas of elevated and minimal risk, accounting for the uncertainty in estimates. Given the heterogeneity in observed site-level DIN estimates paired with uncertainty in model-derived NIP and DON estimates, we have higher confidence in these categorical classifications of DIN over exact estimates. Across our candidate thresholds, the two foci in the Northeast and Upper Midwest originally identified by Diuk-Wasser et al. [11], in addition to a progressively larger area encompassing much of the Upper Midwest and Northeast and spreading further south, were identified as having elevated relative DIN levels. Based on comparison with field-derived estimates, these estimated categories provided good discrimination of areas of true low risk (high negative predictive value) while somewhat overpredicting areas of true high risk (moderate positive predictive value). The lower positive predictive values are expected for organisms currently undergoing range expansion, since they have not occupied their full niche yet [36, 58]. However, species invasion or expansion dynamics are complex, likely involving more than environmental correlates [36, 59]. Climatic or environmental predictors of tick and pathogen distributions have not been consistent across studies and are highly dependent on the data available to build models [60]. Together, these points highlight the need for continued tick surveillance and periodic evaluation and refinement of acarological risk models.

Our method for estimating and presenting NIP was subject to several limitations. We used county-level covariates, which were average long-term weather conditions, as explanatory variables such that covariate values did not directly relate to on-the-ground conditions for any specific collection event. While the fitted variables may correlate with host or tick population distributions or behavior of nymphal ticks [19, 61], we do not attribute causality by any of these fitted relationships given the spatial and temporal averaging we employed; they delineate spatial patterns in observed NIP. Also, we could not include data on host community composition or distributions, factors directly related to maintenance and amplification of pathogen transmission, as these are not available at the county level across the entire eastern USA [60]. We also acknowledge that presenting acarological risk at the county scale masks sub-county-level variation and does not explicitly illustrate changes or stabilization of NIP with expanding tick and pathogen populations. Finally, since the field-derived NIP estimates were not completely independent of the site-level nymphal testing data used to fit the GAM, our evaluations may be biased. However, cross-validation indicated a robust model fit, and the secondary validations with reported presence of *B. burgdorferi* s.s., a truly independent dataset, illustrated high discriminatory ability in classifying low versus moderate–high NIP. With this, we have higher confidence in the relative versus exact magnitudes of modeled NIP.

## Conclusions

In conclusion, we present model-based estimates of county-level NIP and DIN in the eastern USA. Owing to the inherent lack of precision in estimated and measured DIN values, we favored presenting categorical risk estimates (e.g., elevated vs. low acarological risk). However, we note that the choice of where to threshold the model estimates will depend on their intended use and the preferences for maximizing sensitivity or specificity. Additionally, DIN alone is insufficient for estimating Lyme disease risk, as it does not include variation in human encounters with infected nymphs due to behavior or probability of successful pathogen transmission [62]. Our GAM predicted relatively higher NIP in the Upper Midwest and Northeast, with lower estimated prevalence throughout the Southeast and Great Plains regions. Estimated DIN followed this similar spatial distribution, with gradations in the magnitude of DIN throughout northern parts of the eastern USA. The spatial patterns in DIN were similar to those of DON, suggesting that spatial variation in estimated DIN was primarily driven by DON, rather than NIP, as previously suggested [11]. Despite interannual variation in prevalence estimates, prevalence often stabilizes within predictable ranges after the pathogen becomes established, with state-level estimates providing adequate information for most public health purposes [45]. Regions classified as suitable to support high DIN were stable over nearly a quarter-century. Therefore, measuring DON and pathogen presence along the leading edges of colonization could be prioritized by tick surveillance programs over intensively and repeatedly sampling the same sites within known endemic areas. Our results indicate that county-level estimates of DON and NIP derived from non- or partially overlapping sites could provide adequate DIN estimates to characterize broad categories of acarological risk for Lyme disease. Further tick surveillance could be targeted to counties that were categorized as elevated DIN but had reported density estimates of zero, to provide an updated estimate of human encounters with infected host-seeking nymphs. Our current and previously [13] presented risk maps of NIP, DON, and DIN provide another step towards identifying the most predictive metric(s) of human cases of tick-borne diseases.

## Supplementary Information

Additional file 1: Table S1. Fitted parametric and smooth terms in generalized additive model (GAM) of nymphal infection prevalence. Table S2. Mean and range of covariates in the final GAM for counties used in model fitting and across all counties in the eastern United States. Table S3. Moran’s *I* test results for analysis of spatial autocorrelation of site-level residual. Table S4. Contingency tables of estimated and observed relative DIN using four candidate cut points to dichotomize DIN. Table S5. Contingency tables of estimated DIN vs. reported nymphal collections and pathogen circulation using four candidate cut points to dichotomize estimated DIN. Fig. S1. Reference maps for regional naming used in manuscript and modeled suitability range for *Ixodes scapularis* ticks in the eastern United States. Fig. S2. Maps of summarized surveillance intensity metrics for tick surveillance with nymphal testing results reported. Fig. S3. Maps summarizing tick surveillance data in counties included in the evaluation of county-level average NIP. Fig. S4. Reported circulation of *B. burgdorferi *s.s. in *I. scapularis* ticks and collection of host-seeking nymphal *I. scapularis *ticks. Fig. S5. Model diagnostic plots and metrics for fitted GAM. Fig. S6. Pearson residuals from fitted GAM by county and state. Fig. S7. Pearson residuals relative to site- and county-level collection event characteristics. Fig. S8. Map of county-level summaries of Pearson residuals. Fig. S9. Maps of county-level covariate values and multivariate environmental suitability surface (MESS) for covariates included in the final GAM. Fig. S10. Map of county-level categorization of predicted NIP based on ROC curve analysis*. *Fig. S11. Prediction error of modeled entomological metrics (DON and DIN) relative to metrics derived from tick surveillance. Fig. S12. Maps of estimated relative DIN categories relative to evidence of *B. burgdorferi* s.s. and host-seeking *I. scapularis* nymphs.

## Data Availability

The tick surveillance datasets analyzed during the current study are not publicly available because data sharing agreements in ArboNET preclude sharing data below the county spatial scale. Environmental datasets used in analyses are publicly available from the respective agencies (USGS National Land Cover: https://doi.org/10.5066/P9KZCM54; WorldClim: https://www.worldclim.org/data/worldclim21.html).

## Abbreviations

CDC: US Centers for Disease Control and Prevention
GAM: Generalized additive model
DIN: Density of host-seeking *Borrelia burgdorferi* s.s.-infected *Ixodes scapularis* nymphs
DON: Density of host-seeking *Ixodes scapularis* nymphs
NIP: Prevalence of *Borrelia burgdorferi* s.s. in *Ixodes scapularis* nymphs
