# Supplementary material for: Estimated density of Borrelia burgdorferi sensu stricto-infected Ixodes scapularis nymphs in the eastern United States
Source: Parasit Vectors. 2025 Aug 18;18:350. doi: 10.1186/s13071-025-06937-2 (PMC12362874; doi:10.1186/s13071-025-06937-2)
Supplement: Supplementary file 1 — Additional file 1: Table S1. Fitted parametric and smooth terms in generalized additive model (GAM) of nymphal infection prevalence. Table S2. Mean and range of covariates in the final GAM for counties used in model fitting and across all counties in the eastern United States. Table S3. Moran’s I test results for analysis of spatial autocorrelation of site-level residual. Table S4. Contingency tables of estimated and observed relative DIN using four candidate cut points to dichotomize DIN. Table S5. Contingency tables of estimated DIN vs. reported nymphal collections and pathogen circulation using four candidate cut points to dichotomize estimated DIN. Fig. S1. Reference maps for regional naming used in manuscript and modeled suitability range for Ixodes scapularis ticks in the eastern United States. Fig. S2. Maps of summarized surveillance intensity metrics for tick surveillance with nymphal testing results reported. Fig. S3. Maps summarizing tick surveillance data in counties included in the evaluation of county-level average NIP. Fig. S4. Reported circulation of B. burgdorferi s.s. in I. scapularis ticks and collection of host-seeking nymphal I. scapularis ticks. Fig. S5. Model diagnostic plots and metrics for fitted GAM. Fig. S6. Pearson residuals from fitted GAM by county and state. Fig. S7. Pearson residuals relative to site- and county-level collection event characteristics. Fig. S8. Map of county-level summaries of Pearson residuals. Fig. S9. Maps of county-level covariate values and multivariate environmental suitability surface (MESS) for covariates included in the final GAM. Fig. S10. Map of county-level categorization of predicted NIP based on ROC curve analysis. Fig. S11. Prediction error of modeled entomological metrics (DON and DIN) relative to metrics derived from tick surveillance. Fig. S12. Maps of estimated relative DIN categories relative to evidence of B. burgdorferi s.s. and host-seeking I. scapularis nymphs. [file 13071_2025_6937_MOESM1_ESM.pdf]

Additional File 1

In reference to "Estimated density of *Borrelia burgdorferi* sensu stricto-infected *Ixodes scapularis* nymphs in the eastern United States" in *Parasites & Vectors*

Karen M. Holcomb, Erik Foster, Sarah E. Maes, Christina M. Parise, Lynn M. Osikowicz, Andrias Hojgaard, Rebecca J. Eisen

This Additional Material contains 5 tables and 12 figures. The following provides a brief description of each.

- Table S1. Fitted parametric and smooth terms in generalized additive model (GAM) of nymphal infection prevalence.
- Table S2. Mean and range of covariates in the final GAM for counties used in model fitting and across all counties in the eastern United States.
- Table S3. Moran's *I* test results across sets of randomly selected points for analysis of spatial autocorrelation of site-level residual.
- Table S4. Contingency tables of estimated and observed relative DIN using four candidate cut points to dichotomize DIN.
- Table S5. Contingency tables of estimated DIN vs. reported nymphal collections and pathogen circulation using four candidate cut points to dichotomize estimated DIN.
- Figure S1. Reference maps for regional naming used in manuscript and modeled suitability range for *Ixodes scapularis* ticks in the eastern United States.
- Figure S2. Maps of summarized surveillance intensity metrics for tick surveillance with nymphal testing results reported.
- Figure S3. Maps summarizing tick surveillance data in counties included in the evaluation of county-level average NIP.
- Figure S4. Reported circulation of *B. burgdorferi* s.s. in *I. scapularis* ticks and collection of host-seeking nymphal *I. scapularis* ticks.
- Figure S5. Model diagnostic plots and metrics for fitted GAM.
- Figure S6. Pearson residuals from fitted GAM by county and state.
- Figure S7. Pearson residuals from fitted GAM relative to site- and county-level collection event characteristics.
- Figure S8. Map of county-level summaries of Pearson residuals.
- Figure S9. Maps of county-level covariate values and multivariate environmental suitability surface (MESS) for covariates included in the final GAM.
- Figure S10. Map of county-level categorization of predicted NIP based on threshold identified through a ROC curve analysis.
- Figure S11. Prediction error of modeled entomological metrics (DON and DIN) relative to metrics derived from tick surveillance.
- Figure S12. Maps of estimated relative DIN categories relative to evidence of *B. burgdorferi* s.s. and host-seeking *I. scapularis* nymphs.

References

1. U.S. Census Bureau: Regions and Divisions. https://www2.census.gov/geo/pdfs/maps-data/maps/reference/us_regdiv.pdf (2021). Accessed 14 Jun 2024.

2. Hahn MB, Jarnevich CS, Monaghan AJ, Eisen RJ. Modeling the geographic distribution of *Ixodes scapularis* and *Ixodes pacificus* (Acari: Ixodidae) in the contiguous United States. J Med Entomol. 2016;53 5:1176-91; doi: 10.1093/jme/tjw076.
3. Hahn MB, Jarnevich CS, Monaghan AJ, Eisen RJ. Response: The geographic distribution of *Ixodes scapularis* (Acari: Ixodidae) revisited: The importance of assumptions about error balance. J Med Entomol. 2017;54 5:1104-6; doi: 10.1093/jme/tjx096.
4. Burtis JC, Foster E, Schwartz AM, Kugeler KJ, Maes SE, Fleshman AC, et al. Predicting distributions of blacklegged ticks (*Ixodes scapularis*), Lyme disease spirochetes (*Borrelia burgdorferi* sensu stricto) and human Lyme disease cases in the eastern United States. Ticks Tick Borne Dis. 2022;13 5:102000; doi: 10.1016/j.ttbdis.2022.102000.
5. Wood SN. Fast stable restricted maximum likelihood and marginal likelihood estimation of semiparametric generalized linear models. J Roy Stat Soc B Met. 2011;73 1:3-36.
6. Wood SN: *Generalized additive models:An introduction with R*. 2nd edn. New York: Chapman and Hall/CRC; 2017.

Table S1. Fitted parameter estimates and approximate significance of smooth terms in generalized additive model (GAM).

	**Covariate**	**Estimate**	**Standard error**	**z-value**	***P*-value**
Parametric term	Intercept	-1.55	0.04	-42.60	<0.001
		**Est df[a]**	**Ref. df[b]**	χ^2	***P*-value[c]**
Smooth terms	Temperature seasonality (Bio4)	4.94	5.29	29.10	<0.001
	Max. temperature of warmest month (Bio5)	3.05	3.36	93.98	<0.001
	Precipitation of wettest quarter (Bio16)	2.30	3.65	21.65	0.001
	county[d]	245.84	547.00	1061.17	<0.001

Smooth terms of bioclimatic covariates fitted using thin-plate splines and default basis dimension (k = 10). See Fig. 1A for plot of smooth functions for these covariates. GAM model with an adjusted R^2 of 0.324 and 39.1% deviance explained.

[a] Estimated (est.) degrees of freedom (edf) for smooth term.

[b] Reference (ref.) degrees of freedom for smooth term.

[c] *P*-values were computed without considering uncertainty in smoothing parameter estimates so may be somewhat low.

[d] Random effect for county fitted using ridge penalties.

Table S2. Range of covariates considered for describing and predicting *B. burgdorferi* s.s. infection prevalence in *Ixodes scapularis* nymphs. Ranges shown for the 551 counties with observations for training the model and the full 2,694 counties across the eastern United States[a] for which we produced estimates.

**Covariate**	**Mean (range) in included counties**	**Mean (range) across eastern US[a]**
Temperature seasonality[b] (Bio4; °C)	967.4 (648.6-1289.1)	896.5 (331.4-1344.2)
Max temperature of warmest month (Bio5; °C)	28.3 (22.6-34.2)	30.9 (22.6-37.3)
Precipitation of wettest quarter (Bio16; mm)	313.9 (236.0-486.5)	332.5 (130.3-658.0)

[a] States in CONUS that are split by or completely east of the 100th meridian (Additional File 1: Fig. S1).

[b] Standard deviation of monthly temperature, multiplied by 100.

Table S3. Moran's *I* test results of Pearson residuals from ten sets of randomly selected points.

**Set**	**Observed**	**Expected**	**Standard deviation**	***P*-value**
1	-0.0057	-0.0005	0.0017	0.002
2	-0.0023	-0.0005	0.0018	0.317
3	-0.0056	-0.0005	0.0020	0.009
4	-0.0056	-0.0005	0.0017	0.003
5	-0.0053	-0.0005	0.0019	0.011
6	-0.0066	-0.0005	0.0018	< 0.001
7	-0.0053	-0.0005	0.0017	0.006
8	-0.0060	-0.0005	0.0017	0.001
9	-0.0075	-0.0005	0.0019	< 0.001
10	-0.0019	-0.0005	0.0018	0.422

Table S4. Contingency table of observed and predicted relative DIN using the indicated cut point (A-D) to categorize DIN.

A. 25th-percentile of non-zero observed DIN as cut point (1.93/1,000 m^2)				
		Observed relative DIN category		
		Minimal[a]	Elevated[c]	Total
Predicted relative DIN category	Minimal[a]	175	17	192
	Uncertain[b]	68	26	94
	Elevated[c]	108	90	198
	Total	351	133	484
Acc: (175+90)/390 = 67.9%	Sens: 90/107 = 84.1%	Spec: 175/283 = 61.8%	PPV: 90/198 = 45.5%	NPV: 175/192 = 91.1%

B. Cut point to achieve 90% sensitivity (1.60/1,000m^2)				
		Observed relative DIN category		
		Minimal[a]	Elevated[c]	Total
Predicted relative DIN category	Minimal[a]	158	12	170
	Uncertain[b]	59	25	84
	Elevated[c]	125	105	230
	Total	342	142	484
Acc: (158+105)/400 = 65.8%	Sens: 105/117 = 89.7%	Spec: 158/283 = 55.8%	PPV: 105/230 = 45.7%	NPV: 158/170 = 92.9%

C. Cut point to achieve 95% sensitivity (1.03/1,000m^2)				
		Observed relative DIN category		
		Minimal[a]	Elevated[c]	Total
Predicted relative DIN category	Minimal[a]	120	7	127
	Uncertain[b]	64	17	81
	Elevated[c]	148	128	276
	Total	332	152	484
Acc: (120+128)/403 = 61.5%	Sens: 128/135 = 94.8%	Spec: 120/268 = 44.8%	PPV: 128/276 = 46.4%	NPV: 120/127 = 94.5%

D. Minimum observed non-zero DIN as the cut point (0.11/1,000m^2)				
		Observed relative DIN category		
		Minimal[a]	Elevated[c]	Total
Predicted relative DIN category	Minimal[a]	44	0	44
	Uncertain[b]	41	1	42
	Elevated[c]	221	177	398
	Total	306	178	484
Acc: (44+177)/442 = 50.0%	Sens: 177/177 = 100%	Spec: 44/265 = 16.6%	PPV: 177/398 = 44.5%	NPV: 44/44 = 100%

Observed relative DIN based on county DIN estimates calculated from tick surveillance data and predicted relative DIN based on 95% CI. See Fig. 4 for map of relative DIN categories for counties in the eastern United States. Last row of each table presents diagnostic metrics calculated using counties predicted as either minimal or elevated, not uncertain. Acc: accuracy; Sens: sensitivity; Spec: specificity; PPV: positive predictive value; NPV: negative predictive value.

[a] Minimal relative DIN: upper DIN 95% CI bound < cut point (predicted) or observed DIN < cut point.

[b] Uncertain relative DIN: DIN 95% CI contains cut point.

[c] Elevated relative DIN: lower DIN 95% CI bound ≥ cut point (predicted) or observed DIN ≥ cut point.

Table S5. Contingency table of reported nymphal collections and pathogen presence vs. predicted relative DIN using indicated cut points (A-D) to dichotomize predicted DIN.

A. 25th-percentile of non-zero observed DIN as cut point (1.93/1,000 m^2)

		Reported circulation of pathogen and/or collection of host-seeking nymphs				
		Neither	Pathogen	Nymphs	Both	Total
Predicted relative DIN category	Minimal[a]	1,766	68	127	105	2,066
	Uncertain[b]	116	10	54	85	265
	Elevated[c]	62	22	58	221	363
	Total	1,944	100	239	411	2,694

Acc: (1766+221)/2154 = 92.2% | Sens: 221/326 = 67.8% | Spec: 1766/1828 = 96.6% | PPV: 221/283 = 78.1% | NPV: 1766/1871 = 94.4%

B. Cut point to achieve 90% sensitivity for relative DIN (1.60/1,000 m^2)

		Reported circulation of pathogen and/or collection of host-seeking nymphs				
		Neither	Pathogen	Nymphs	Both	Total
Predicted relative DIN category	Minimal[a]	1,733	60	112	93	1,998
	Uncertain[b]	130	16	52	60	258
	Elevated[c]	81	24	75	258	438
	Total	1,944	100	239	411	2,694

Acc: (1733+258)/2165 = 92.0% | Sens: 258/351 = 73.5% | Spec: 1733/1814 = 95.5% | PPV: 258/339 = 76.1% | NPV: 1733/1826 = 94.9%

C. Cut point to achieve 95% sensitivity for relative DIN (1.03/1,000 m^2)

		Reported circulation of pathogen and/or collection of host-seeking nymphs				
		Neither	Pathogen	Nymphs	Both	Total
Predicted relative DIN category	Minimal[a]	1,635	49	80	66	1,830
	Uncertain[b]	178	19	59	45	301
	Elevated[c]	131	32	100	300	563
	Total	1,944	100	239	411	2,694

Acc: (1635+300)/2132 = 90.8% | Sens: 300/366 = 82.0% | Spec: 1635/1766 = 92.6% | PPV: 300/431 = 69.6% | NPV: 1635/1701 = 96.1%

D. Minimum observed non-zero DIN as cut point (0.11/1,000 m^2)

		Reported circulation of pathogen and/or collection of host-seeking nymphs				
		Neither	Pathogen	Nymphs	Both	Total
Predicted relative DIN category	Minimal[a]	1,017	2	18	1	1,038
	Uncertain[b]	494	12	27	8	541
	Elevated[c]	433	86	194	402	1,115
	Total	1,944	100	239	411	2,694

Acc: (1017+402)/1853 = 76.6% | Sens: 402/403 = 99.8% | Spec: 1017/1450 = 70.1% | PPV: 402/835 = 48.1% | NPV: 1017/1018 = 99.9%

Reported circulation of *B. burgdorferi* s.s. in any life stage of *I. scapularis* and collection of host-seeking *I. scapularis* nymphs by drag/flag sampling obtained from tick surveillance data. Predicted relative DIN categorization based on 95% CI. See Fig. 4 for map of relative DIN categories and Additional File 1: Fig. S4 for map of reported pathogen circulation and collection of host-seeking nymphs. Last row of each table presents diagnostic metrics calculated using counties predicted as either minimal or elevated and with neither or both reported pathogen circulation and collection of host-seeking nymphs. Acc: accuracy; Sens: sensitivity; Spec: specificity; PPV: positive predictive value; NPV: negative predictive value.

[a] Minimal relative DIN: upper DIN 95% CI bound < cut point.

[b] Uncertain relative DIN: DIN 95% CI contains cut point.

[c] Elevated relative DIN: lower DIN 95% CI bound ≥ cut point.

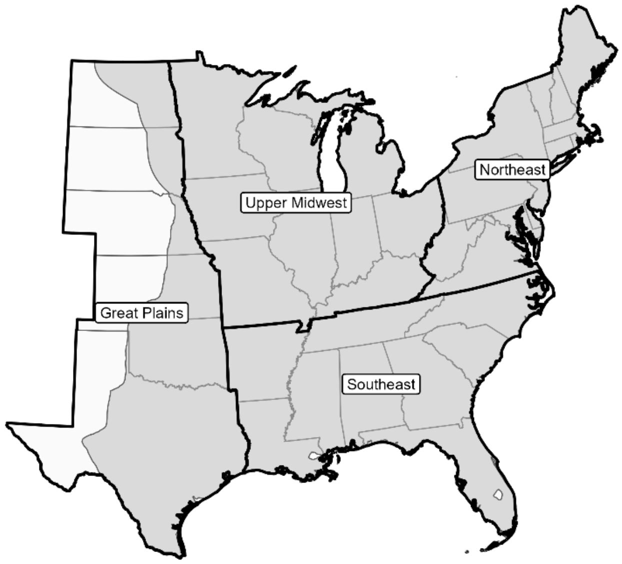

Figure S1. Geographic regions and estimated suitable distribution for *I. scapularis* in the eastern United States. Thick borders denote geographic-based regional naming scheme modified from the U.S. Census Bureau [1]. Thin lines denote state boundaries. Estimated environmentally suitable range for *I. scapularis* ticks (grey shading) generalized from habitat suitability modeling [2, 3, 4].

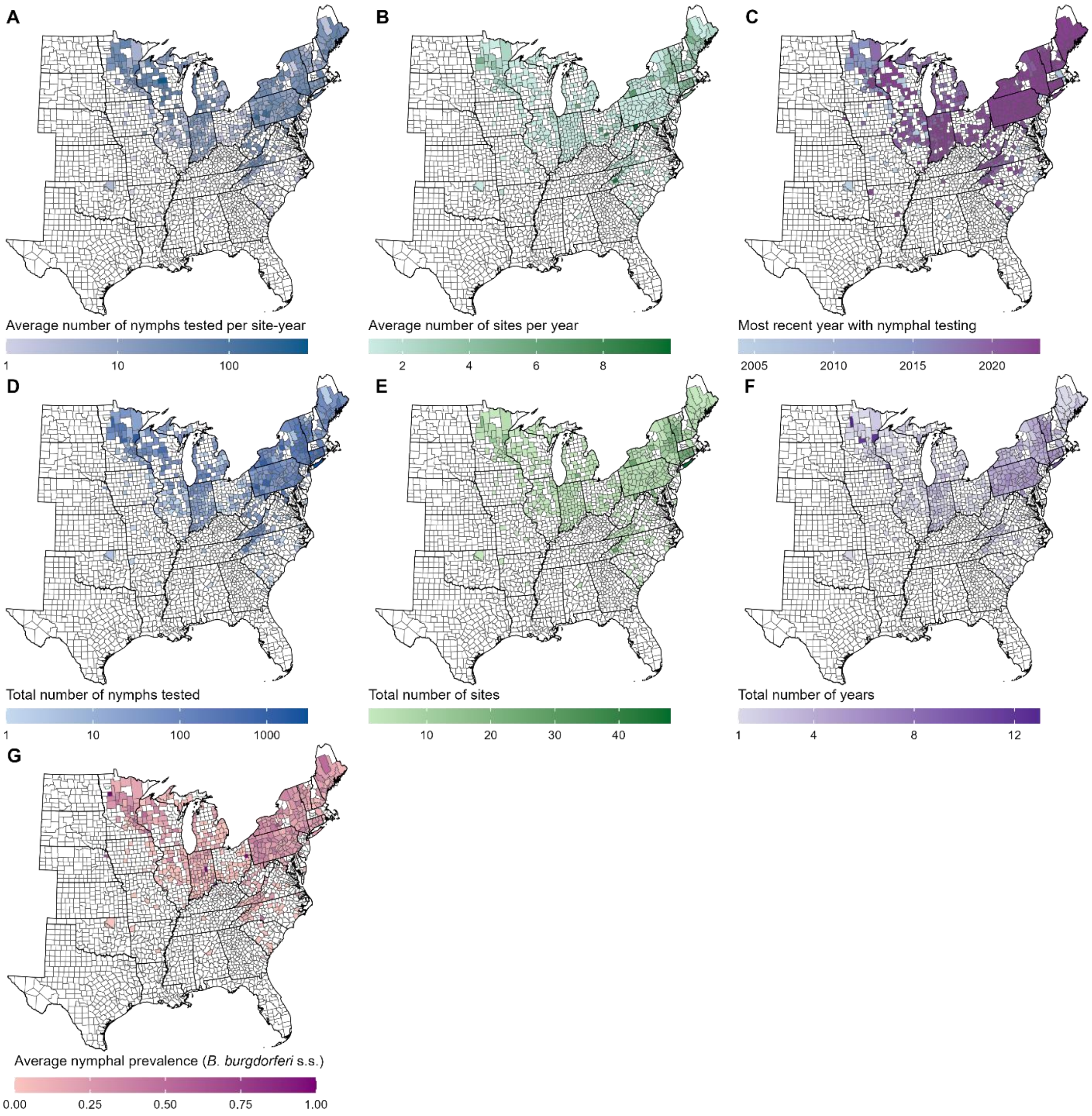

Figure S2. County-level summaries of surveillance of nymphal *I. scapularis* infection prevalence with *B. burgdorferi* s.s. (2004-2023). Surveillance effort metrics include A) average number of nymphs tested per site-year, B) average sites with testing data per year, C) most recent year with nymphal testing data, D) total number of nymphs tested across all years, E) total number of site-years with testing data, and F) number of years with testing per county. F) County-all years estimate of average nymphal infection prevalence. Calculation of prevalence estimates based on site-years with testing results for any number of nymphs (i.e., not using the 25-nymph threshold), but otherwise following the calculation method outlined in the Methods. In all maps, white counties did not have nymphal *B. burgdorferi* s.s. testing data reported to the ArboNET Tick Module.

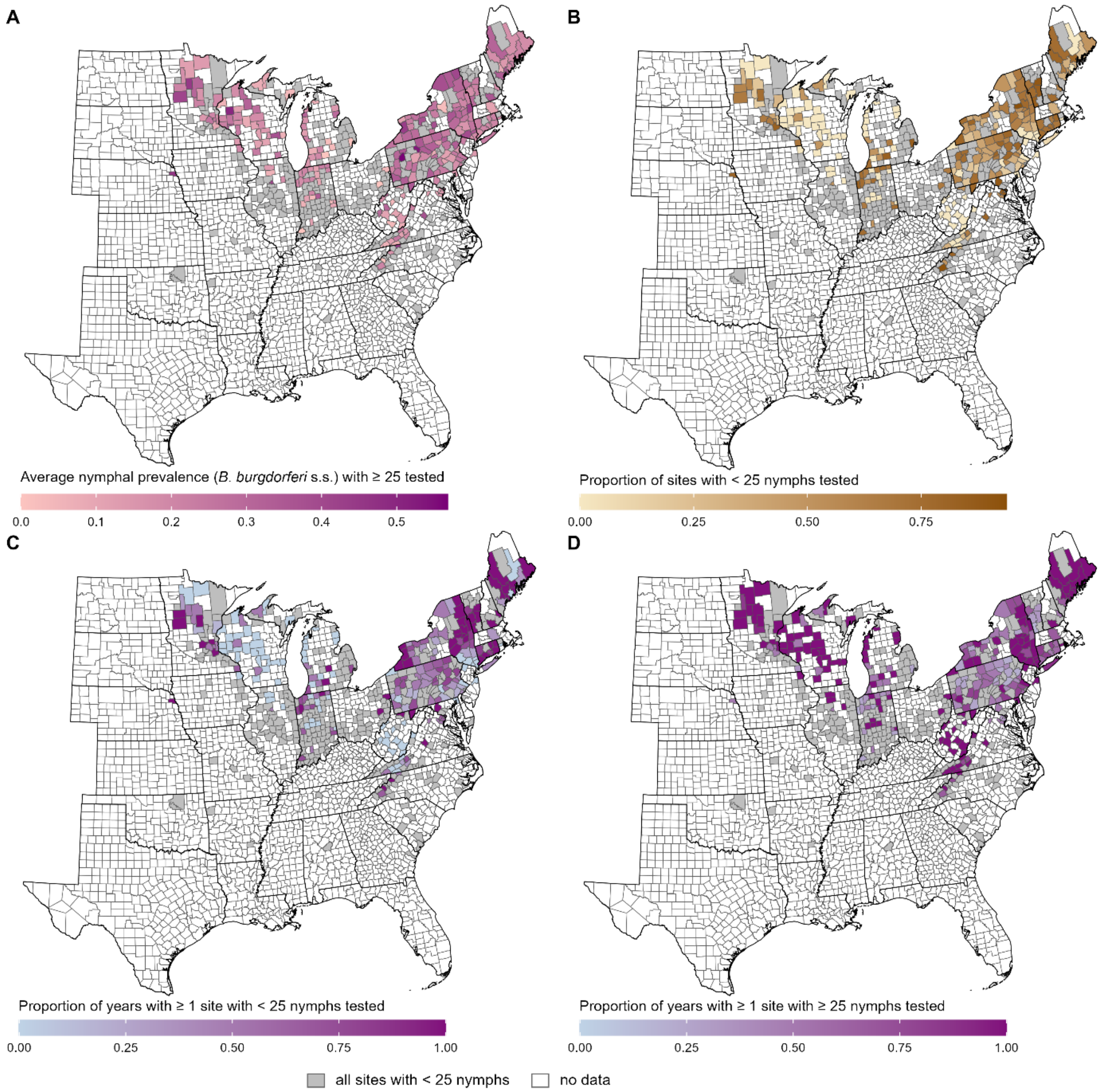

Figure S3. Summaries of tick surveillance data in the 243 counties included in the evaluation of estimated NIP. A) County-all years estimate of average nymphal infection prevalence based on sites with ≥25 nymphs tested. See Methods for details on the calculation method and compare with Additional File 1: Fig. S2G for average prevalence using all site-level data. B-D) Summarization of training data in only the counties used for evaluation of modeled NIP to investigate overlap between datasets. B) Proportion of all sites with < 25 nymphs tested. Proportion of years for which ≥1 site had C) < 25 or D) ≥25 nymphs tested. Note that C) and D) are not mutually exclusive. See Additional File 1: Fig. S2F for plot of total number of years of reported data per county. In all plots, grey counties had reported nymphal testing data in the ArboNET Tick Module, but all sites had < 25 nymphs tested (i.e., only in training data set).

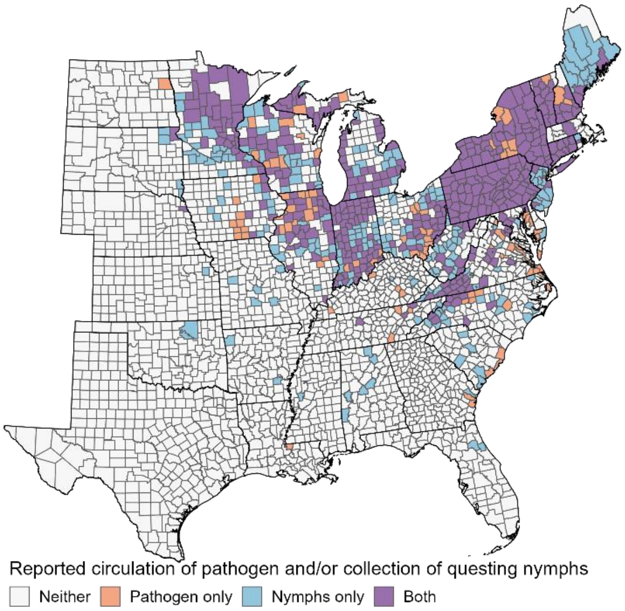

Figure S4. Reported circulation of *B. burgdorferi* s.s. in any *I. scapularis* life stage and collection of host-seeking *I. scapularis* nymphs in the ArboNET Tick Module. Collection of host-seeking nymphs defined as drag or flag sampling resulting in ≥1 *I. scapularis* nymph collected. Counties with no reported collections either had drag/flag sampling resulting in zero nymphs collected or did not report any drag/flag sampling.

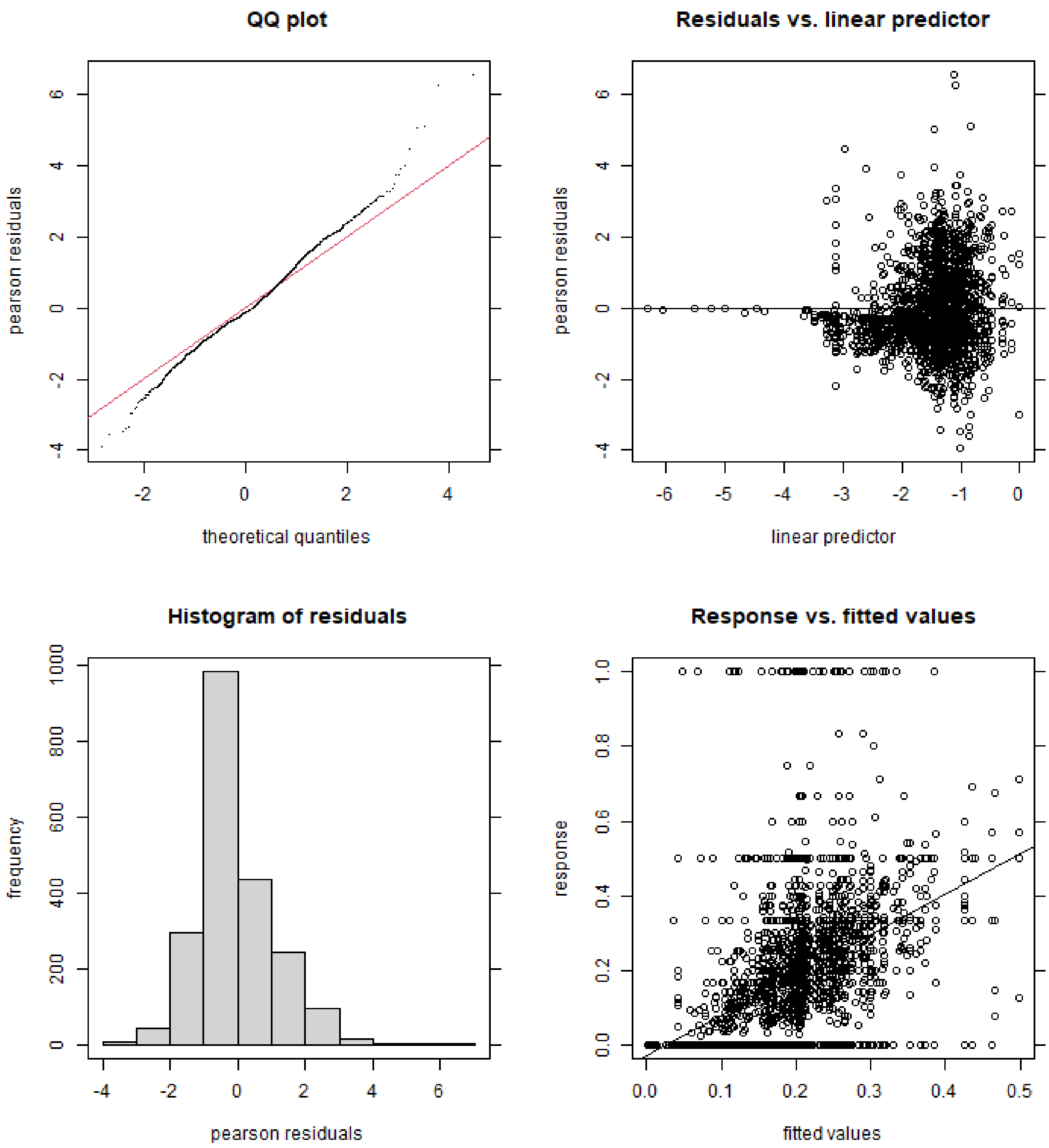

Figure S5. Model diagnostic plots for fitted generalized additive model of nymphal prevalence. Four residual plots produced by the gam.check function in the *mgcv* R package [5, 6] using Pearson residuals. Note that in scatterplots, each point corresponds to a site-level testing result. Diagonal line in response vs. fitted scatterplot indicates 1:1 relationship. Additional diagnostic information produced by the function further indicates good model fit: Hessian positive definite, full model rank (579/579), and basis dimension checks confirmed dimensions not too low (all thin-plate splines with k-index > 1.0 and *P*-values of 0.99-1.00).

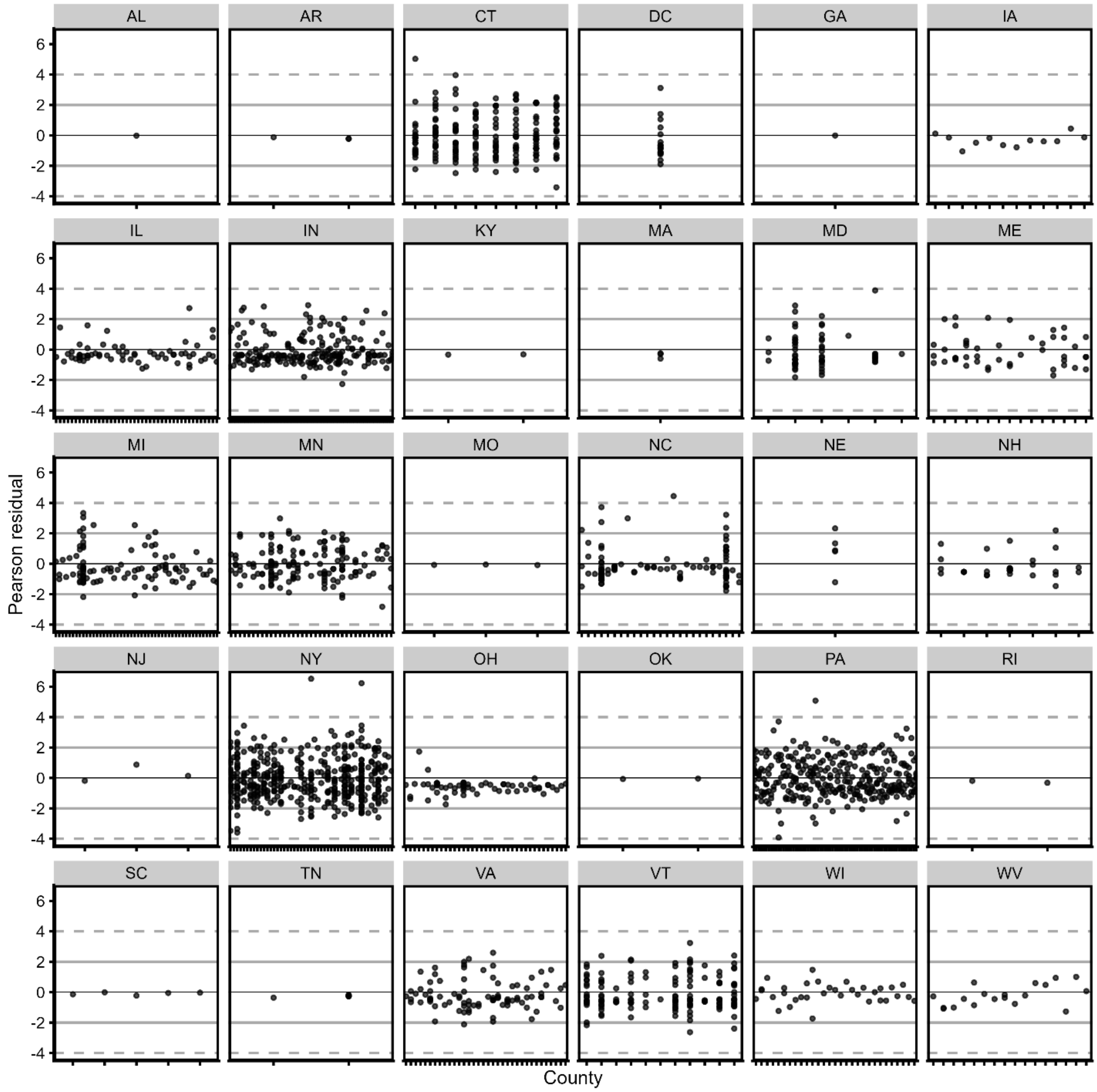

Figure S6. Pearson residual from fitted generalized additive model (GAM) by county and state. Each point corresponds to an individual site. Panels labeled by two-letter state abbreviations. Tick marks along the x-axis in each panel represents unique counties with testing nymphal tick testing data included in the model. County names or abbreviations not given due to space. See Additional File 1: Fig. S8 for maps of summarized Pearson residuals by county. Horizontal grey lines indicate residuals of magnitude >2 ("high" residuals, solid) and >4 ("extreme" residuals, dashed).

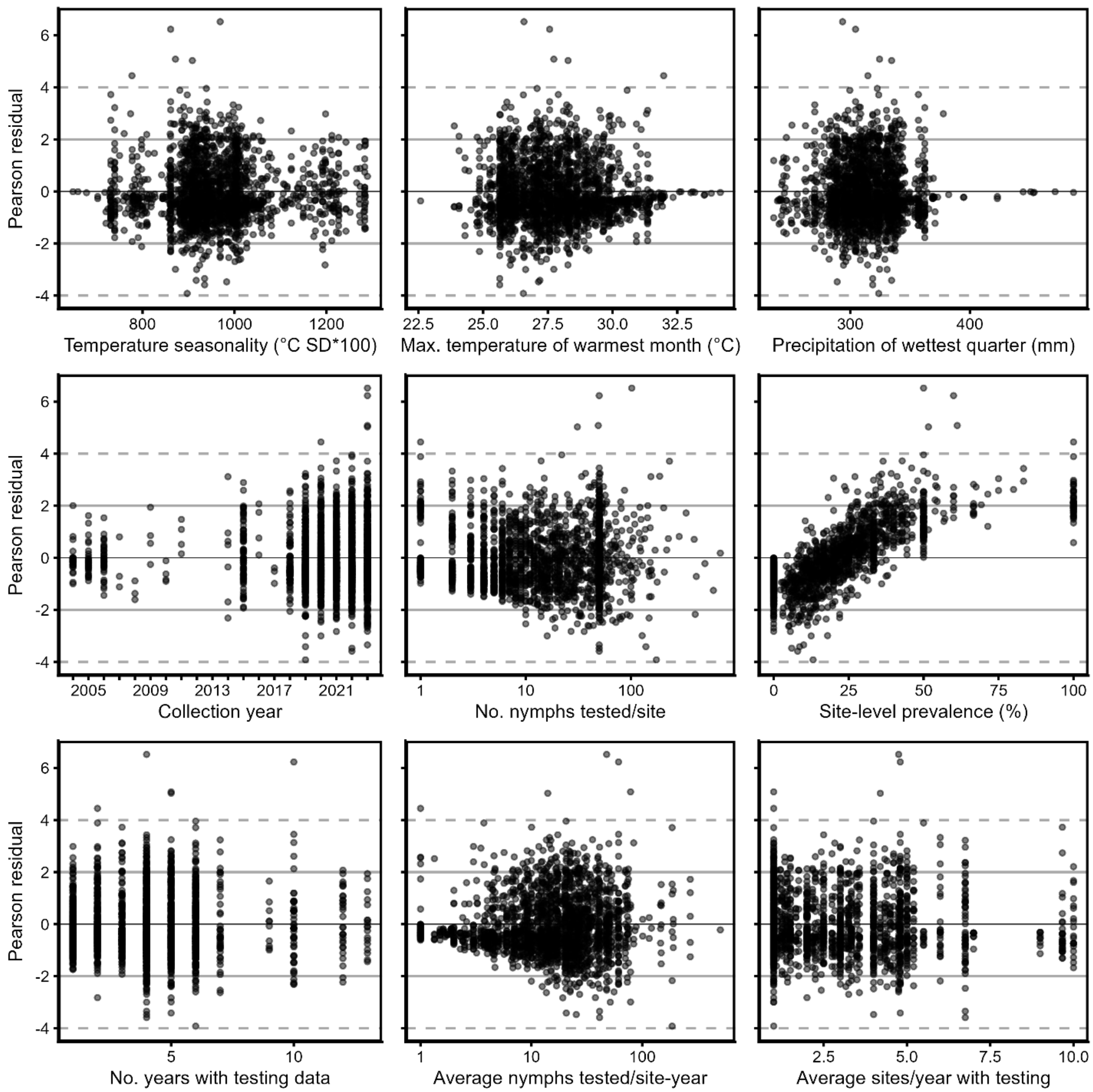

Figure S7. Additional investigations of Pearson residuals from fitted generalized additive model (GAM). Each point corresponds to an individual site. The top two rows present residuals vs. bioclimatic covariates (top row) or collection attributes (middle row) incorporated into the GAM. Collection year informed model weights and number of nymphs tested was used as the model offset. The bottom row presents residuals vs. surveillance intensity metrics calculated at the county-all year scale. See Additional File 1: Fig. S2 for maps of surveillance intensity metrics. Horizontal grey lines indicate residuals of magnitude >2 (“high” residuals, solid) and >4 (“extreme” residuals, dashed).

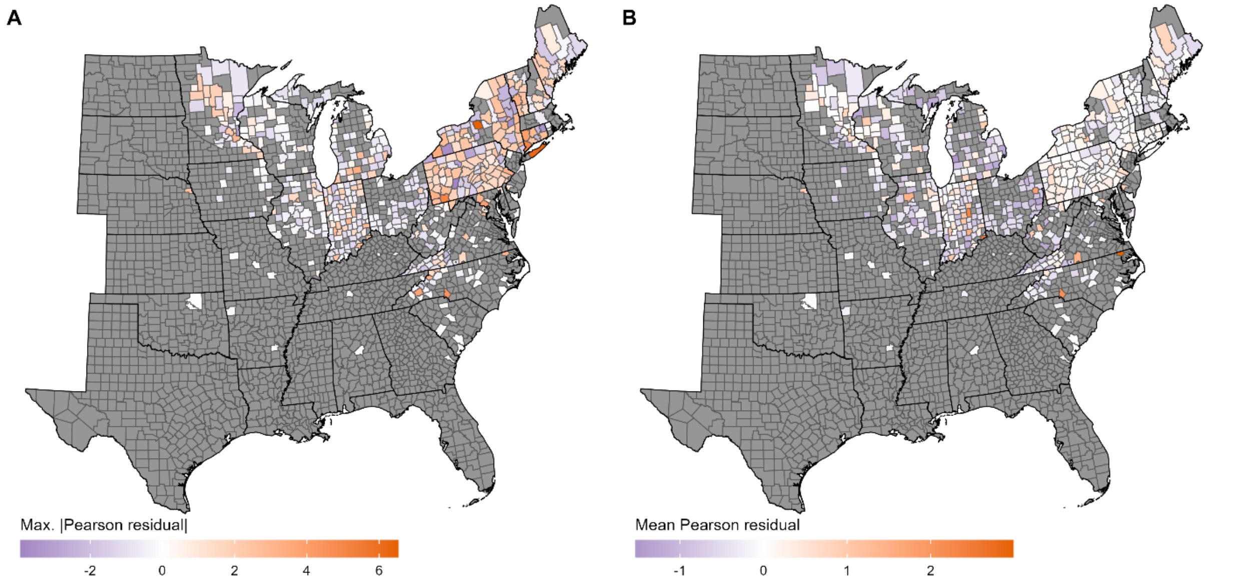

Figure S8. County-level summaries of Pearson residuals from fitted generalized additive model (GAM). A) Maximum magnitude of and B) average site-level residual per county. See Additional File 1: Figs. S6-S7 for scatterplots of site-level residuals.

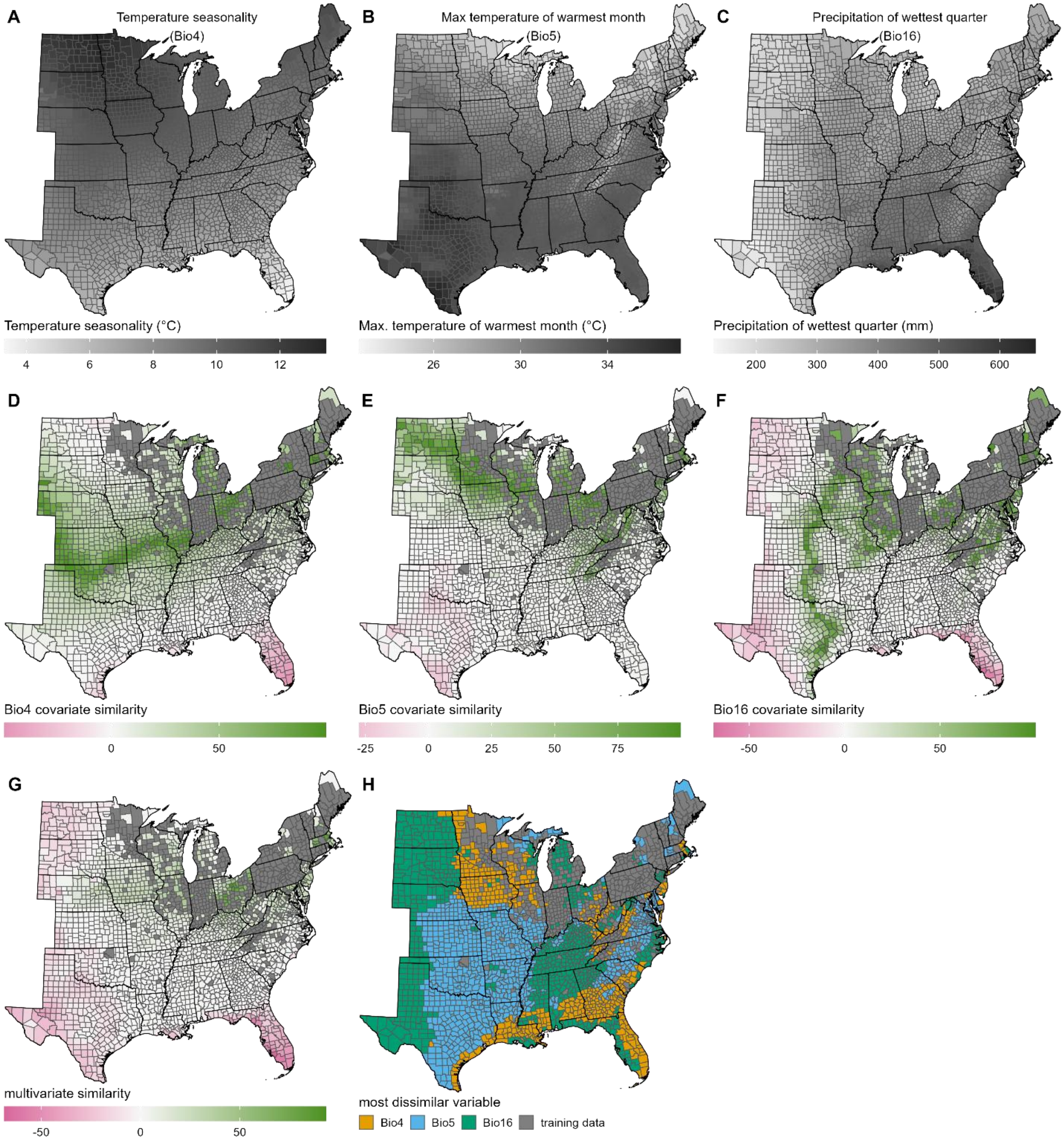

Figure S9. Covariate values and multivariate environmental suitability surface (MESS). A-C) County-level covariates in final fitted generalized additive model (GAM). Note values for Bio4 divided by 100 for interpretation (see Table 1 for details on derivation, Additional File 1: Table S2 for numerical values). D-F) Univariate similarity for each covariate as measured by Euclidean distance from the center of the distribution in the training data (grey counties). G) Multivariate similarity as the minimum similarity across all variables and the H) covariate with this minimum similarity. In D-G), green shading (positive similarity) indicates the covariate falls within the distribution in the training data with darker shade representing closer to the center of the distribution. Pink shading (negative similarity) represents values outside the training distribution with darker shading representing larger distances and more dissimilar conditions. White (similarity = 0) indicates values at the min. or max. of the training distribution.

A

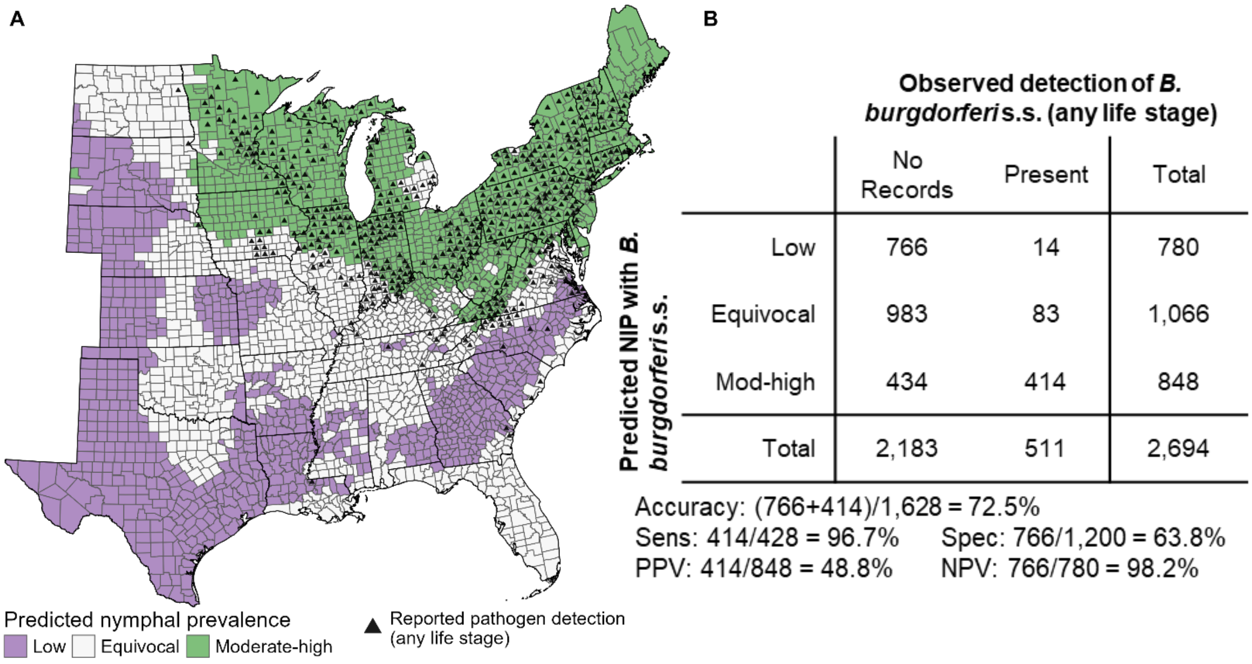

B

Predicted NIP with *B. burgdorferi* s.s.	Observed detection of *B. burgdorferi* s.s. (any life stage): No Records	Present	Total
Low	766	14	780
Equivocal	983	83	1,066
Mod-high	434	414	848
Total	2,183	511	2,694

Accuracy: (766+414)/1,628 = 72.5%
Sens: 414/428 = 96.7% Spec: 766/1,200 = 63.8%
PPV: 414/848 = 48.8% NPV: 766/780 = 98.2%

Figure S10. Categorization of predicted nymphal prevalence based on ROC curve analysis with reported detection of *B. burgdorferi* s.s. Threshold of 8.16% predicted prevalence identified as balancing sensitivity and specificity. Predicted prevalence categorized using 95% credible intervals (CrI) such that counties categorized as low had upper 95% CrI bound < 8.16%, equivocal had 8.16% include in 95% CrI, and moderate-high (mod-high) had lower 95% CrI bound > 8.2%. A) Map of county-level predicted NIP and reported detections of *B. burgdorferi* s.s. in any *I. scapularis* life stage. B) Contingency table of predicted and observed detection status. Last row in table presents diagnostic metrics calculated using counties predicted as either low or moderate-high, not equivocal. Acc: accuracy; Sens: sensitivity; Spec: specificity; PPV: positive predictive value; NPV: negative predictive value.

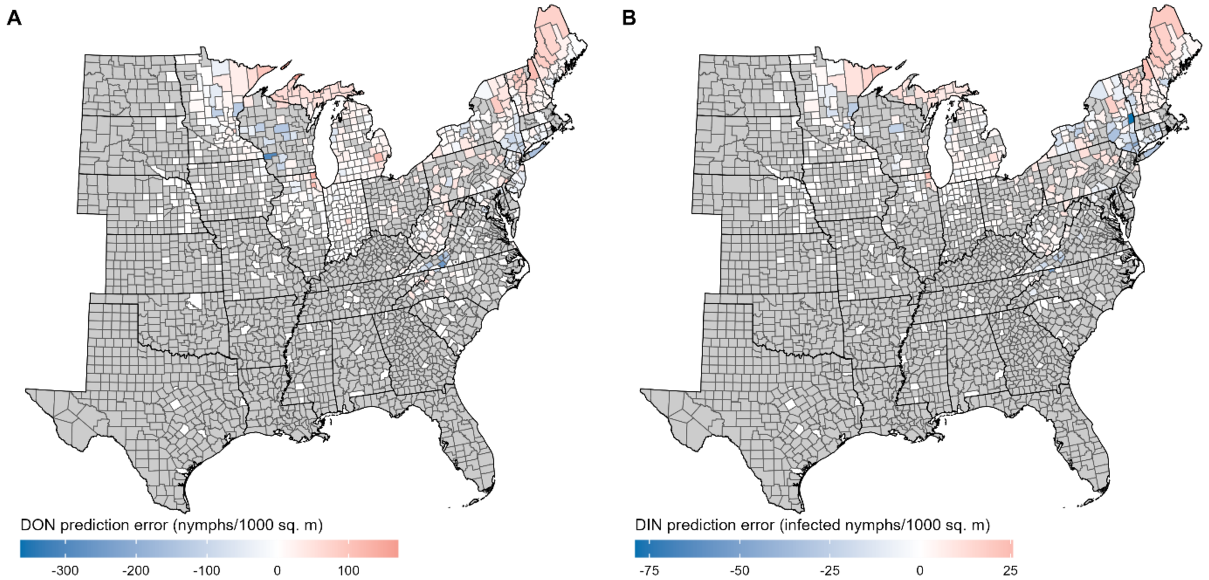

Figure S11. Error in predicted A) density of host-seeking *I. scapularis* nymphs (DON), and B) density of *B. burgdorferi* s.s-infected *I. scapularis* nymphs (DIN). Observed data for calculating prediction error derived from reported tick surveillance to the ArboNET Tick Module. See Fig. 1E for prediction error in nymphal infection prevalence (NIP).

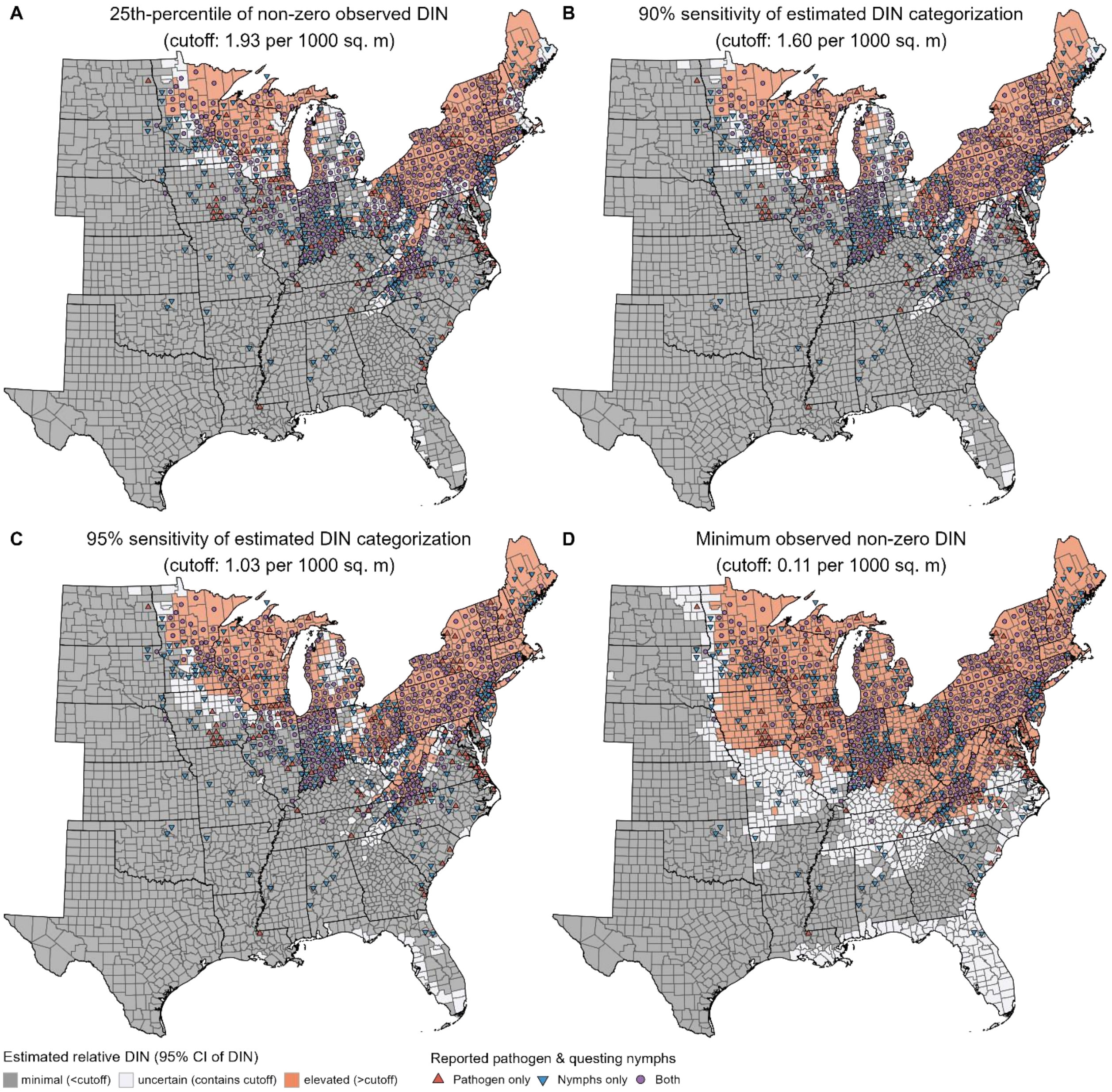

Figure S12. Estimated relative DIN categories relative to evidence of *B. burgdorferi* s.s. and host-seeking *I. scapularis* nymphs. Detection of *B. burgdorferi* s.s. (pathogen) in any life stage of *I. scapularis* ticks and collection of host-seeking *I. scapularis* nymphs through drag or flag sampling reported to the ArboNET Tick Module (see Additional File 1: Fig. S4). A-D) Categorization of estimated DIN using 95% CI and indicated candidate cut points. Minimal relative DIN (grey shading) classified when the upper DIN 95% CI bound < cut point. Uncertain relative DIN (white shading) classified when the DIN 95% CI contained cut point. Elevated relative DIN (coral shading) classified when the lower DIN 95% CI bound ≥ cut point. See Additional File 1: Table S5 for calculation of diagnostic metrics for each categorization.
